# Molecular Ecological Basis of Grasshopper (*Oedaleus asiaticus*) Phenotypic Plasticity under Environmental Selection

**DOI:** 10.3389/fphys.2017.00770

**Published:** 2017-10-10

**Authors:** Xinghu Qin, Kun Hao, Jingchuan Ma, Xunbing Huang, Xiongbing Tu, Md. Panna Ali, Barry R. Pittendrigh, Guangchun Cao, Guangjun Wang, Xiangqun Nong, Douglas W. Whitman, Zehua Zhang

**Affiliations:** ^1^State Key Laboratory for Biology of Plant Diseases and Insect Pests, Institute of Plant Protection, Chinese Academy of Agricultural Sciences, Beijing, China; ^2^School of Biology, University of St. Andrews, St. Andrews, United Kingdom; ^3^Scientific Observation and Experimental Station of Pests in Xilingol Rangeland, Ministry of Agriculture, Institute of Plant Protection, Chinese Academy of Agricultural Sciences, Xilinhot, China; ^4^Entomology Division, Bangladesh Rice Research Institute, Dhaka, Bangladesh; ^5^Department of Entomology, Michigan State University, East Lansing, MI, United States; ^6^School of Biological Sciences, Illinois State University, Normal, IL, United States

**Keywords:** *Oedaleus asiaticus*, phenotypic plasticity, environmental variation, transcriptome, eco-transcriptomic architecture

## Abstract

While ecological adaptation in insects can be reflected by plasticity of phenotype, determining the causes and molecular mechanisms for phenotypic plasticity (PP) remains a crucial and still difficult question in ecology, especially where control of insect pests is involved. *Oedaleus asiaticus* is one of the most dominant pests in the Inner Mongolia steppe and represents an excellent system to study phenotypic plasticity. To better understand ecological factors affecting grasshopper phenotypic plasticity and its molecular control, we conducted a full transcriptional screening of *O. asiaticus* grasshoppers reared in four different grassland patches in Inner Mongolia. Grasshoppers showed different degrees of PP associated with unique gene expressions and different habitat plant community compositions. Grasshopper performance variables were susceptible to habitat environment conditions and closely associated with plant architectures. Intriguingly, eco-transcriptome analysis revealed five potential candidate genes playing important roles in grasshopper performance, with gene expression closely relating to PP and plant community factors. By linking the grasshopper performances to gene profiles and ecological factors using canonical regression, we first demonstrated the eco-transcriptomic architecture (ETA) of grasshopper phenotypic traits (ETAGPTs). ETAGPTs revealed plant food type, plant density, coverage, and height were the main ecological factors influencing PP, while insect cuticle protein (ICP), negative elongation factor A (NELFA), and lactase-phlorizin hydrolase (LCT) were the key genes associated with PP. Our study gives a clear picture of gene-environment interaction in the formation and maintenance of PP and enriches our understanding of the transcriptional events underlying molecular control of rapid phenotypic plasticity associated with environmental variability. The findings of this study may also provide new targets for pest control and highlight the significance of ecological management practice on grassland conservation.

## Introduction

A fundamental question in evolutionary biology is how phenotypic variation is created within a population. This topic is important because phenotypic variation is a raw resource for selection (Whitman and Agrawal, 2009); that is, the environment selects among phenotypes. Variation in phenotype is a critical component of the selection process (Whitman and Ananthakrishnan, 2009). Phenotypic variation derives mostly from two sources: genetic variation and phenotypic plasticity (PP) (Harrelson and Valentino, 2013; Lea, 2017; Ziv et al., 2017). Additionally, selection can result in extremely rapid evolution of a species as well as evolutionary changes over small spatial scales (Carroll et al., 2007; Baythavong, 2011; Richardson et al., 2014; Real, 2017). Selection can hypothetically occur in minutes as when a sudden, extreme environmental event instantly eliminates all individuals not phenotypically and genetically resistant to the lethal factor. As such, the rapid creation of phenotypic diversity via phenotypic plasticity is of great importance not only both for individual fitness and survival but also for evolutionary biology.

Evolution in PP has been thought of as resulting from variable natural selection in ecologically diverse environments, and it has been demonstrated experimentally that plasticity mediates the adaptive expression of phenotypes in nature (Agrawal, 2001; Scheiner and DeWitt, 2004; Dayan, 2016; Chevin and Hoffmann, 2017). The expression of PP, as arising from genome and genome-wide transcriptomes in different conditions, is the response and adaptation of organisms to diverse ecological environments (López-Maury et al., 2008; Reuter et al., 2017). Factors initiating PP include stimuli and cues that change initial hatchling size, growth rates, nutrient titers, development time, body size, and so forth (Whitman and Ananthakrishnan, 2009; Corona et al., 2016). Phenotypic plasticity may also create more phenotypic variation than mutation itself (Kokko et al., 2017). Not only is it extremely rapid, but it also appears to be constant and continuous throughout the lives of individuals (Whitman and Agrawal, 2009; Pfab et al., 2016; Chevin and Hoffmann, 2017; Kokko et al., 2017). Timeframes for these phenotypic changes can range from seconds (e.g., homeostasis, behavior, and some instantaneous color changes), to minutes (e.g., some forms of acclimation, and induction of some defense and detoxifying enzymes), and to months or longer, often with profound, and sometimes relatively short term or immediate fitness consequences. Thus, populations undergoing phenotypic plasticity are not only moving targets for selection, but different individuals within the population may be changing phenotypes in different directions, creating an ever-changing diversity of phenotypes, upon which multiple selective factors can act (Whitman and Ananthakrishnan, 2009; Corona et al., 2016).

Adaptive PP enables organisms to maximize their fitness in response to environmental heterogeneity (Baythavong, 2011; Kokko et al., 2017). However, it is clear that different habitats can induce diverse phenotypes and that this process can be extremely rapid—i.e., within hours or days. Does this rapid, sometimes within-generation, creation of phenotypic diversity play an important role in evolution? This is an important question with many caveats. However, one critical of this aforementioned question involves understanding the mechanism by which habitat change induces phenotype change. On a broader scale, molecular mechanisms associated with PP in organisms were, until recently, largely concealed (Aubin-Horth and Renn, 2009; Zhu, 2016; Gao et al., 2017). Fortunately, modern advances in molecular biology and informatics (such as classic QTL analysis, RAD sequencing, transcriptomics, proteomics, etc.) allow us for the first time to begin to understand the molecular machinery that interfaces environment and genotype and that translates environmental signals into altered phenotypes (Aubin-Horth and Renn, 2009 Harrelson and Valentino, 2013; Wang and Kang, 2014; Zhu, 2016; Gao et al., 2017). More recently, an eco-transcriptome architecture method, employed to identify and quantify genes and environmental factors responsible for phenotypic traits, has come into use in molecular ecology as a way to interpret complex ecological and molecular relationships related to phenotypic traits (Mank, 2017; Takahashi, 2017). This offers a great opportunity to add to our understanding of the molecular underpinnings of PP.

In this paper, we take a well-known rangeland pest, *O. asiaticus* Bey-Bienko, one of the most abundant grasshoppers in the Mongolian plateau (Li et al., 1990), as a model organism for investigating the origin of PP and its molecular basis. Although the steppe grasslands of Inner Mongolia appear, at first glance, to be relatively uniform, in reality, they present a spatially and temporally dynamic mosaic of environmental conditions that include slope, exposure, soil composition and moisture, and relative humidity (RH), as well as plant community-composition, density, maturity, architecture, and percentage of ground cover. At present, we do not know how this environmental variability influences grasshopper performance, population dynamics, and the degree of vegetation destruction by this pest insect. Specifically, how and how quickly do small environmental changes alter phenotypes, and how do the resulting phenotypic changes influence grasshopper survival, fitness, population dynamics of this pest insect? Previous studies show that certain stimuli, such as temperature, light, food, social interactions, and others, can induce PP in grasshoppers, including changes in physiology, development, morphology, behavior, fecundity, life-history, and pest status (Hodin, 2009; Musolin and Saulich, 2012; Pfab et al., 2016; Simões et al., 2016; García-Navas et al., 2017). How these function for *O. asiaticus* remains unknown.

In order to make clear the ecological factors inducing PP and its molecular basis, we reared *O. asiaticus* grasshoppers in four different grassland types common within the Inner Mongolian grassland ecosystem dominated by *Stipa krylovii* Roshev., *Leymus chinensis* (Trin.) Tzvel., *Cleistogenes squarrosa* (Trin.) Keng., and *Artemisia frigida* Willd, respectively. We investigated the PP in grasshopper performance, including resulting changes to size, mass, development rate, survival, and other measures. We then analyzed the environmental factors and transcriptomic changes that could act on PP. We linked changes in gene expression to specific enzymes, biochemical pathways, and performance variables, in order to gain a comprehensive molecule-to-whole organism understanding of the complex and interconnected biochemical mechanisms underlying PP, including how environment influences transcriptomic changes, and how transcriptomic changes influence ultimate individual fitness. Our study allowed us to address, in the Discussion, six fundamental questions around gene and environment-wide associated PP: What factors induce PP? How liable are organisms to exhibit PP? How pervasive is PP in individuals? What are the molecular mechanisms that facilitate PP? What is the spatial-geographic component of PP? Is PP positively related to stress?

## Results

### Habitat conditions

The four patches [*C. squarrosa* type (**Cs**), *L. chinensis* type (**Lc**), *S. krylovii* type (**Sk**), and *A. frigida* type (**Af**)] differed significantly in numerous vegetative traits (Table 1), including dominant plant species coverage. Based on Simpson's Diversity Index, Sk, and Af had significantly higher plant diversity compared to Cs and Lc (*F* = 23.14, df = 18, *P* < 0.0001). But no difference was found between Sk and Af. Lc had significantly lower plant diversity and Sk higher plant diversity among all the four patches based on Shannon-Weiner Index (*F* = 16.24, *df* = 19, *P* < 0.0001). Vegetation coverage was highest in Lc, which was similar to Cs (*F* = 1.93, df = 19, *P* = 0.1651). Vegetation height was highest in Sk, which was significantly different from Cs, Lc, and Af (*F* = 63.56, df = 19, *P* < 0.0001). Vegetation density was significantly higher in Lc, than in all other vegetative types (*F* = 111.69, df = 19, *P* < 0.0001). Lc showed the highest biomass, followed by Af, Cs, and Sk (*F* = 3.72, df = 19, *P* = 0.0333) (Table 1).

**Table 1:** Patch conditions of *O. asiaticus* in different patches.

**Patches**	**Sk**	**Cs**	**Lc**	**Af**
Plant Simpson's diversity index	0.95 ± 0.004a	0.72 ± 0.06b	0.54 ± 0.04c	0.93 ± 0.02a
Plant Shannon-Weiner index	1.72 ± 0.15a	1.01 ± 0.30b	0.068 ± 0.041c	0.72 ± 0.06b
Average grass coverage (%)	49 ± 8bc	66 ± 5ab	83 ± 10a	41 ± 8c
Average grass height (cm)	66 ± 3a	22 ± 2c	31 ± 2b	32 ± 3b
Average vegetation density (stems/m^2^)	88 ± 26b	51 ± 7b	639 ± 47a	55 ± 5b
Average vegetation dry biomass (g/m^2^)	98.8 ± 10b	102.5 ± 7b	220.8 ± 57a	167.1 ± 14ab

*Patch plant community conditions including plant diversity, coverage, biomass, plant density and height are shown in the table. Sk, Cs, Lc, and Af indicate patches dominated by Stipa krylovii, Cleistogenes squarrosa, Leymus chinensis, Artemisia frigida, respectively. Table values indicate mean±SEM. Those marked by lowercase letters are significantly different within each row based on Tukey's Studentized Range (HSD) at P < 0.05*.

### Grasshopper feeding preferences

In the laboratory feeding trials, *O. asiaticus* expressed strong feeding preferences. Based on the Selective Index (*SI*), the grasshoppers preferred Sk > Cs > Lc > Af (Table 2). The grasshoppers consumed a limited amount of *A. frigida* (Family Asteraceae) and instead consumed larger proportions of three grasses present in the test cages. There was no significant difference between male and female in *SI*, when they were reared separately. *SI* of Cs and Sk were significantly higher than Lc and Af (*F* = 9.94, df = 19, *P* = 0.0006), but there were no significant differences between Cs and Sk (*P* = 0.4138) or between Lc and Af (*P* = 0.367).

**Table 2:** Grasshopper performance in different patches.

**Patches**	**Sk**	**Cs**	**Lc**	**Af**
Plant selective index	1.63 ± 0.20a	1.39 ± 0.21a	0.50 ± 0.28b	0.18 ± 0.19b
Survival rate	0.37 ± 0.026a	0.45 ± 0.055a	0.44 ± 0.066a	0.16 ± 0.099b
Development time (d)	21.2 ± 0.37b	20.4 ± 0.25b	22.8 ± 0.67a	23 ± 0.45a
Female body mass (mg)	598.7 ± 59.4ab	624.5 ± 23.9a	466.3 ± 38.7b	586.7 ± 55.9ab
Female body length (mm)	28.0 ± 0.8ab	28.7 ± 0.6a	26.5 ± 0.3b	27.2 ± 0.8ab
Female relative growth rate (%/d)	3.98 ± 0.91a	3.72 ± 0.74a	3.82 ± 0.36a	1.88 ± 0.88a
Female overall performance	11.6 ± 1.7a	12.4 ± 0.7a	12.7 ± 1.0a	5.4 ± 2.6b

*Sk, Cs, Lc, and Af indicate patches dominated by Stipa krylovii, Cleistogenes squarrosa, Leymus chinensis, Artemisia frigida, respectively. Table values indicate mean±SEM. Those marked by lowercase letters are significantly different within each row based on Tukey's Studentized Range (HSD) at P < 0.05*.

### Grasshopper performance in four treatments

Considering the strong feeding preferences of *O. asiaticus* for certain plant species and that the availability of favored food plants varied among the four plots (Table 1), it is not surprising that grasshopper performance differed significantly among the four patches (Table 2). Af and Lc were the least-preferred plant species, and, concomitantly, grasshopper performance (survival, development time, body mass, body length, growth rate, and overall performance) was lowest in patches dominated by those plants (Table 2). Survival rates were significantly lower in the Af habitat (*F* = 4.09, df = 19, *P* = 0.0263). In this habitat, the grasshopper mostly refused to feed on plants in the composite family, which accounts for the largest proportion in Af patches. In fact, according to our observation and estimation, grasshoppers were forced to eat *A. frigida*. In two Af replicates, all of the grasshoppers had died by Day 27, suggesting that Af-dominated grassland is generally unsuitable to live for *O. asiaticus* grasshoppers.

Grasshoppers in the Af and Lc patches had significantly longer development times compared to Cs and Sk patches (*F* = 7.54, df = 19, *P* = 0.0023). Body length (*F* = 10.74, df = 18, *P* = 0.0044) and body mass (*F* = 8.82, df = 18, *P* = 0.0086) were lowest in Lc, and significantly lower than in Cs, but not significantly different compared to Sk and Af (Table 2). The lowest grasshopper growth rate was observed in the Af habitat, although growth rates did not differ significantly among the patches (*F* = 1.71, df = 19, *P* = 0.205) (Table 2). Overall grasshopper performance (relative growth rate × survival rate, *F* = 4.13, df = 18, *P* = 0.0254, Table 2) was lowest in Af, and significantly lower than in the other treatments.

### Reference transcriptome assembly and annotation

Illumina Sequencing of *O. asiaticus* adult female whole body yielded over 41,669,258 clean reads out of 45,950,081 raw reads per sample (Supplementary Table 1), and 152,789,985 nucleotides (transcripts) (Supplementary Table 2). A set of 178,711 transcripts and 144,883 unigenes were generated for which the counts for N50 were 1,900 and 1,313, respectively (Supplementary Tables 2, 3). As expected, half of the sequences annotated to NCBI non-redundant protein sequences were matched to insect species, of which the most abundant were: *Zootermopsis nevadensis* (22.7%), *Stegodyphus mimosarum* (6.9%), *Tribolium castaneum* (6.3%), *Acyrthosiphon pisum* (5.0%) (Supplementary Figure 1). Additionally, 43,939 (30.32%) unigenes were successfully annotated through BLAST searches in the seven indicated databases; most of the unigenes (33,604) annotated were from the non-redundant (NR) database, whereas the fewest (4,481) were from nucleotide database (NT) (Supplementary Table 4). In clusters of orthologous groups of proteins (KOG), 13,958 annotated genes were assigned to 26 groups. The (R) general functional prediction only, (T) signal transduction, and (O) post-translational modification, protein turnover, and chaperone groups contained the greatest number of annotated genes (4,012, 1,641, and 464 genes, respectively) (Supplementary Figure 2). Furthermore, 9,268 genes were annotated with pathways in KEGG. Most of the genes were annotated with the signal transduction, translation, and carbohydrate metabolism categories (945, 779, and 628 genes, respectively) (Supplementary Figure 3). The transcriptome data of *O. asiaticus* females was submitted to SRA database in NCBI (ID: SRP059063).

### Comparison of transcript expression levels of *O. asiaticus* with different performance

**Figure 1:**
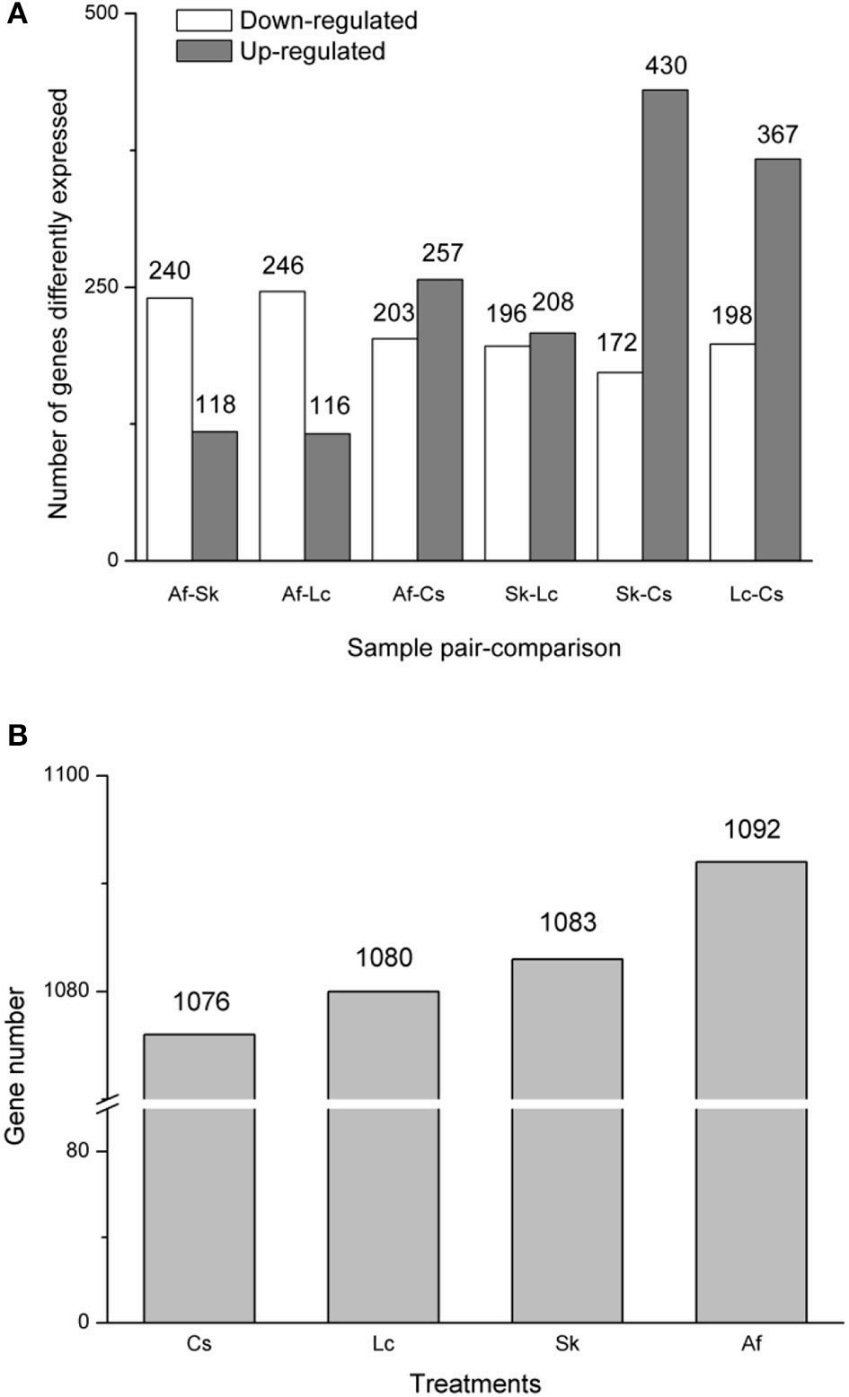
**(A)** The distribution of differentially expressed genes (DEGs). The X-axis indicates treatment-vs.-treatment, while the Y-axis indicates the number of the DEGs. The gray bar indicates up-regulated genes, while the white bar indicates down-regulated genes. **(B)** The total numbers of differently expressed genes. All genes were at *q* <0.005, |log2.Fold_change|>1.

Gene expression levels differed among the four treatments, as shown by FPKM (fragments per kilobase per million mapped reads). The FPKM density distribution patterns were similar among the four samples; they presented one peak near zero. Grasshoppers in the Lc treatment showed the highest density (higher expression rate), followed by Af, Cs, and Sk (Supplementary Figure 4). The numbers of differentially expressed (*q* < 0.005, |log2.Fold_change|>1) genes for each pairwise comparison among treatments were: Af vs. Sk: 358 (240 down-regulated, 118 up-regulated); Af vs. Lc: 362 (246 down-regulated, 116 up-regulated); Af vs. Cs: 460 (203 down-regulated, 257 up-regulated); Sk vs. Lc: 404 (196 down-regulated, 208 up-regulated): Sk vs. Cs 602 (172 down-regulated, 430 up-regulated); Lc vs. Cs: 565 (198 down-regulated, 367 up-regulated) (Figure 1A). Af had the highest number of differently expressed genes, followed by Sk, Lc, and Cs (Figure 1B).

To identify genes involved in habitat-response, specific gene expression patterns that tended to correlate with grasshopper survival rates and overall performance were generated by K-means cluster. Across all four treatments, 39 genes increased their levels of expression as overall performance decreased (Supplementary Figure 5A). These genes include darpin, hexamerin-like protein 4, protein lethal/crystalline, and hemolymph protein, etc. (Supplementary Datasheet 1—Table 1). In contrast, 78 genes decreased their expression as overall performance decreased (Supplementary Figure 5B), including “suppressor of forked protein,” “heat shock protein 20.7,” “major allergen Per a 1.0101,” “cellulose,” etc. (Supplementary Datasheet 1—Table 2). To investigate their biological functions, all the differentially expressed genes were mapped to 265 pathways in the KEGG database. As a result, over 64 pathways were substantially enriched (*P* < 0.05) between different treatments, including galactose metabolism, amino sugar and nucleotide sugar metabolism, metabolic pathways, glycan degradation, and tryptophan metabolism (Supplementary Figure 3; Supplementary Table 5; Supplementary datasheets 2–10).

We also attempted to link grasshopper performance with gene expression by identifying specific genes involved in growth and development. Compared to higher survival rates patches (Cs, Lc, Sk), the up-regulated transcripts in the treatment with the lowest survival rate (Af) included fatty acid synthase (FASN), stearoyl-CoA desaturase (SCD), lactase-phlorizin hydrolase (LCT), and heat shock protein Hsp (Supplementary Table 5). Down-regulated genes included UDP glucose 6-dehydrogenase (UGDH), RAC serine/threonine-protein kinase (AKT), and aspartate aminotransferase (GOT) (Supplementary Table 5; Supplementary Datasheets 6, 9, 10). However, distinct from other higher survival rate patches (Lc, Sk), up-regulated genes—including crystalline (CRY/CRYAB), molecular chaperone HtpG (HSP90A), elongation of very long chain fatty acids protein (ELOVL7), and oligosaccharyl transferase complex (SWP/OST/RPN)—were enhanced highly in the treatment with the highest survival rate (Cs) (Supplementary Table 5).

### KEGG pathway enrichment analysis

KEGG analysis uncovered important transcriptional responses and enzymatic pathways that were influenced differently by the environmental variation. LCT, malZ, SCD, FASN, and HEXA genes were repeatedly enriched in biological pathways such as galactose metabolism, amino sugar, and nucleotide sugar metabolism in Af treatment (Supplementary Table 5; Supplementary Datasheets 2–4). Meanwhile, “amino sugar and nucleotide sugar metabolism and protein processing in endoplasmic reticulum” pathways were repeatedly down-graded in Af (Supplementary Table 5. Af vs. Lc down, Af vs. Sk down, Af vs. Cs down). These lower-level biological and molecular processes may underlie the longer development time and low relative growth rate in Af (Table 2). In contrast, all of these pathways, as well as others as given in Supplementary Table 5 were highly enriched in the higher preforming grasshoppers. In addition, the down-regulated RAC serine/threonine-protein kinase (AKT), which is involved in PI3K-Akt signaling pathway, VEGF signaling pathway, TNF signaling pathway and apoptosis, was down-regulated in Af (Supplementary Table 5, Af vs. Lc down; Supplementary Datasheet 9). Meanwhile, environmentally induced genes “Hsp90,” “Hsp70” etc., showed relatively high over-expression in Cs (Supplementary Table 5; Supplementary Datasheet 6).

### Gene ontology annotation

**Figure 2:**
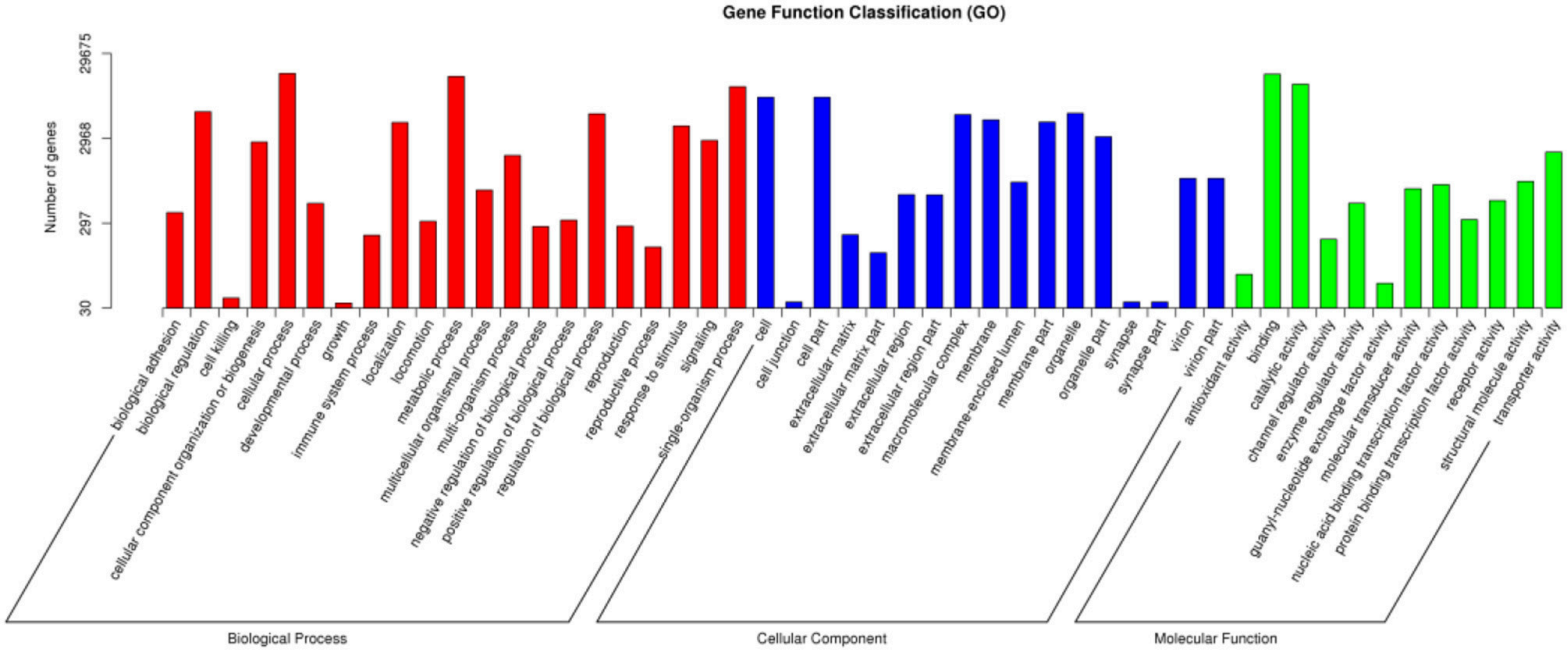
Gene function classification via GO annotation. The differentially expressed genes are grouped into three hierarchically structured GO terms: biological process, cellular component, and molecular function. The y-axis indicates the number of genes in each GO term.

A total of 29,675 unigenes of *O. asiaticus* were subcategorized into fifty hierarchically structured GO classes (Figure 2). These transcripts were categorized as a biological process (47.97%), cellular component (29.38%), or molecular function (22.65%). Among the biological process assignments, a high percentage (22.60%) were assigned to cellular processes. The cellular component terms showed a significant percentage of genes assigned to cell part (19.30%), while molecular functions were associated predominantly with binding (46.86%) (Figure 2).

The chitin metabolic, glucosamine-containing compound metabolic, chitin binding, and amino sugar metabolic processes were the most enriched GO processes across all treatments (Supplementary Table 6). In contrast to low survival patch (Af), high numbers of enriched genes were assigned mainly either for extracellular regions in cellular components (Supplementary Table 6; Af vs. Lc, 38 genes) and hydrolase activity in molecular function (Supplementary Table 6; Af vs. Sk, 74 genes; Af vs. Cs, 96 genes). Furthermore, compared to grasshoppers in high survival patches (Cs, Sk), highly enriched genes similarly assigned for hydrolase activity in molecular function (Supplementary Table 6, Cs vs. Lc, 113 genes), catalytic activity in molecular function (Supplementary Table 6; Cs vs. Sk, 247 genes), and extracellular region in cellular component (Supplementary Table 6; Lc vs. Sk, 56 genes). However, among these, many stress-related genes were enriched differently in the harsh, low-performance patches vs. the favorable, high-performance treatments, including carbohydrate metabolic, proteolysis, single-organism developmental process, and chitinase activity (Supplementary Table 6, Af vs. Lc, Af vs. Sk, Af vs. Cs). These gene-ontology differences suggest molecular level to larger pathway interactions, including cellular and performance PP.

### Association between body plasticity, plant community structure and gene expression in grasshoppers

Across all four treatments, female body length and body mass negatively correlated with plant density (for body length *N* = 36, *r* = −0.56, *P* = 0.0211, body mass *N* = 36, *r* = −0.54, *P* = 0.0154). Hence, less dense patches with ample bare ground and opulent sunshine produced larger, heavier grasshoppers. Developmental time showed a negative correlation as determined by the Shannon-Weiner index (*N* = 20, *r* = −0.48, *P* = 0.0304), possibly because higher-diversity plants produced more secondary compounds that slowed grasshopper development. Conversely, grasshoppers in higher plant density (*N* = 20, *r* = 0.43, *P* = 0.0579), and higher plant biomass (*N* = 20, *r* = 0.43, *P* = 0.0582) patches showed a longer development time, suggesting that sparse, sunny habitats benefited the grasshopper development.

**Figure 3:**
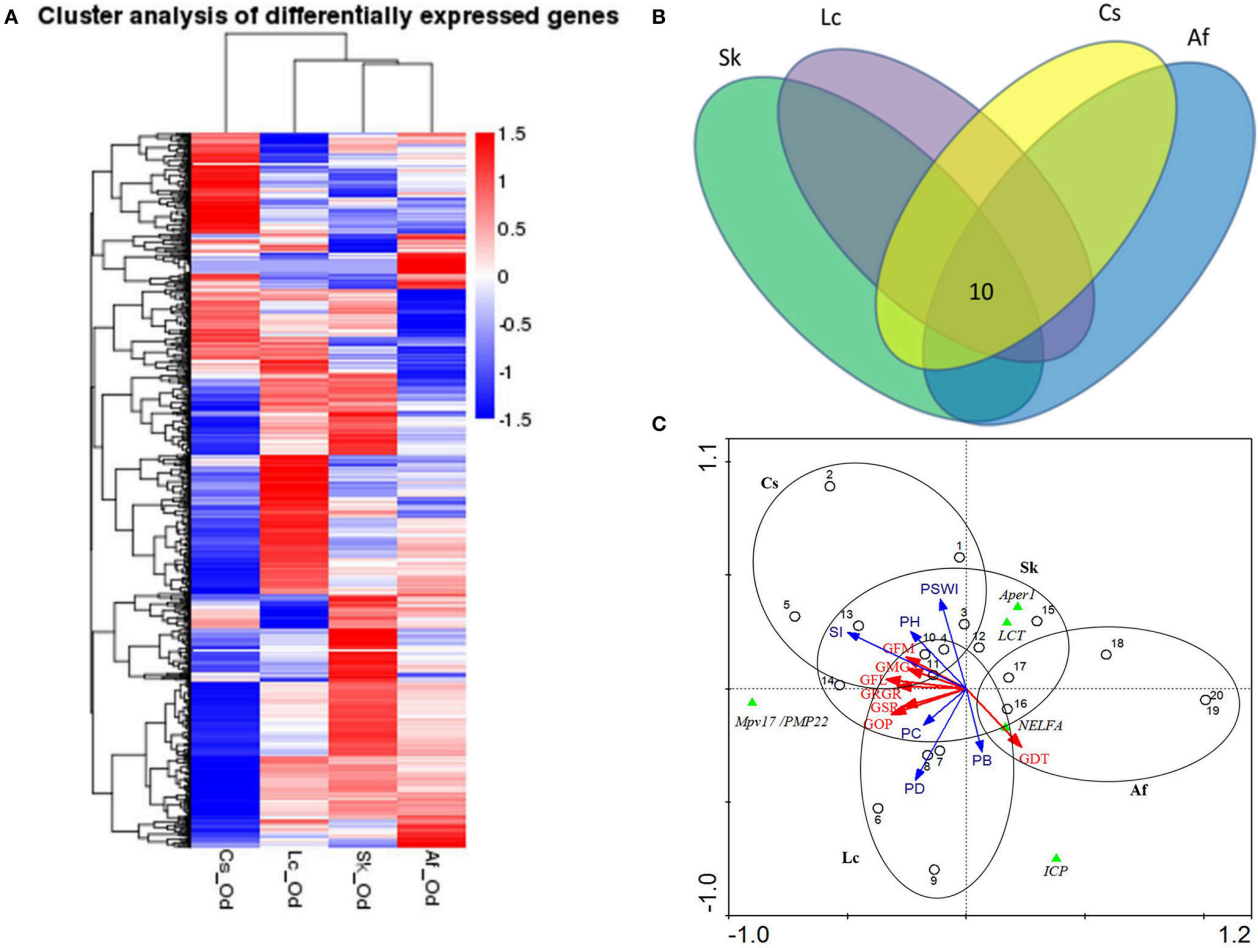
**(A)** Cluster analysis of differentially expressed genes between different samples. The red color indicates the higher expressed genes, while the blue color indicates the lower expressed genes per treatment, and white indicates no difference. The value of the color indicates log_10_(FPKM+1). All genes were at *q*<0.005, |log2.Fold_change|>1. **(B)** The common differentially expressed genes across four patches. Different ovals refer to different patches, where the number indicates the genes shared in all habitats. All genes were at *q*<0.005, FPKM>0.5, |log2.Fold_change|>1. **(C)** The ordination triplot of grasshopper-genes-plant communities. Red arrows indicate grasshopper performance while blue arrows indicate plant community structure. Green triangles indicate the genes, dots indicate samples, and ellipses indicate the four patches, respectively. GFM, grasshopper female mass; GRGR, grasshopper relative growth rate; GMG, grasshoppers' mass gain; GFL, grasshopper female length; GDT, grasshopper development time; GSR, grasshopper survival rate; GOP, grasshopper overall performance; PSWI, plant Shannon-Wiener index; SI, plant selective index; PH, plant height; PD, plant density; PB, plant biomass; PC, plant coverage; Aper1, peritrophin-1; Mpv17/PMP22, the 22 kDa peroxisomal membrane protein; ICP, insect cuticle protein; NELFA, negative elongation factor A; LCT, lactase-phlorizin hydrolase.

Grasshoppers in different patches showed unique gene expression patterns (Figure 3A), with five candidate genes orderly distributed along the two axes. Samples that clustered adjacently coincided with corresponding survival rates, indicating that gene expression patterns lead to differences in performance. This suggests that the construction of an eco-transcriptomic architecture for phenotypic traits is reliable here. Similarly, gene differential expression analysis showed that 10 differentially expressed transcripts were shared among different patches (Figure 3B), but only five of their associated genes could be successfully annotated using the current databases. These were: insect cuticle protein (ICP), peritrophin-1 (Aper-1), lactase-phlorizin hydrolase (LCT), Mpv17/PMP22, and negative elongation factor A (NELFA) (Table 3).

**Table 3:** Differentially expressed genes (DEGs) shared with four patches.

**Gene ID**	**Description**	**Function description**	**Metabolism pathway**
c82685_g1	Insect cuticle protein	Structural constituent of cuticle	–
c63539_g2	Peritrophin-1	Chitin binding Peritrophin-A domain	Chitin binding; chitin metabolic process
c77868_g1	lactase-phlorizin hydrolase (LCT)	Hydrolase activity, hydrolyzing O-glycosyl compounds//hydrolase activity, acting on glycosyl bonds	Carbohydrate metabolic process
c78664_g1	The 22 kDa peroxisomal membrane protein (Mpv17 / PMP22)	Involved in pore-forming activity and may contribute to the unspecific permeability of the organelle membrane	–
c80521_g2	Negative elongation factor A like	Essential component of the NELF complex, a complex that negatively regulates the elongation of transcription by RNA polymerase II. Has an essential role in postembryonic development	–

*All genes' q < 0.005, |log2.Fold_change|>1, FPKM> 0.5*.

We mapped the grasshopper-genes-plant community relations by means of an ordination triplot (Figure 3C). In line with the above, grasshopper performance was mainly explained by (i) the plant intrinsic functional trait—food quality for grasshopper (food selective index, *SI)*, (ii) the plant structural properties—plant density (PD) and plant coverage (PC). The first axis explained 92.9% of species/environment relations (with environment accounting for 74.8% correlations). Adding the second axis, this explained 96.0% of the species/environment relations (of which, 79.5% accounts for the correlation) (Figure 3C). Based on these evidences, we concluded that grasshopper PP was highly subject to habitat plant community structure. From the grasshopper-genes-plant communities (Figure 3C), the results show that expression levels of LCT, Aper-1, and NELFA negatively related to overall grasshopper performance, while expression of Mpv17/PMP22 positively related to overall performance.

**Figure 4:**
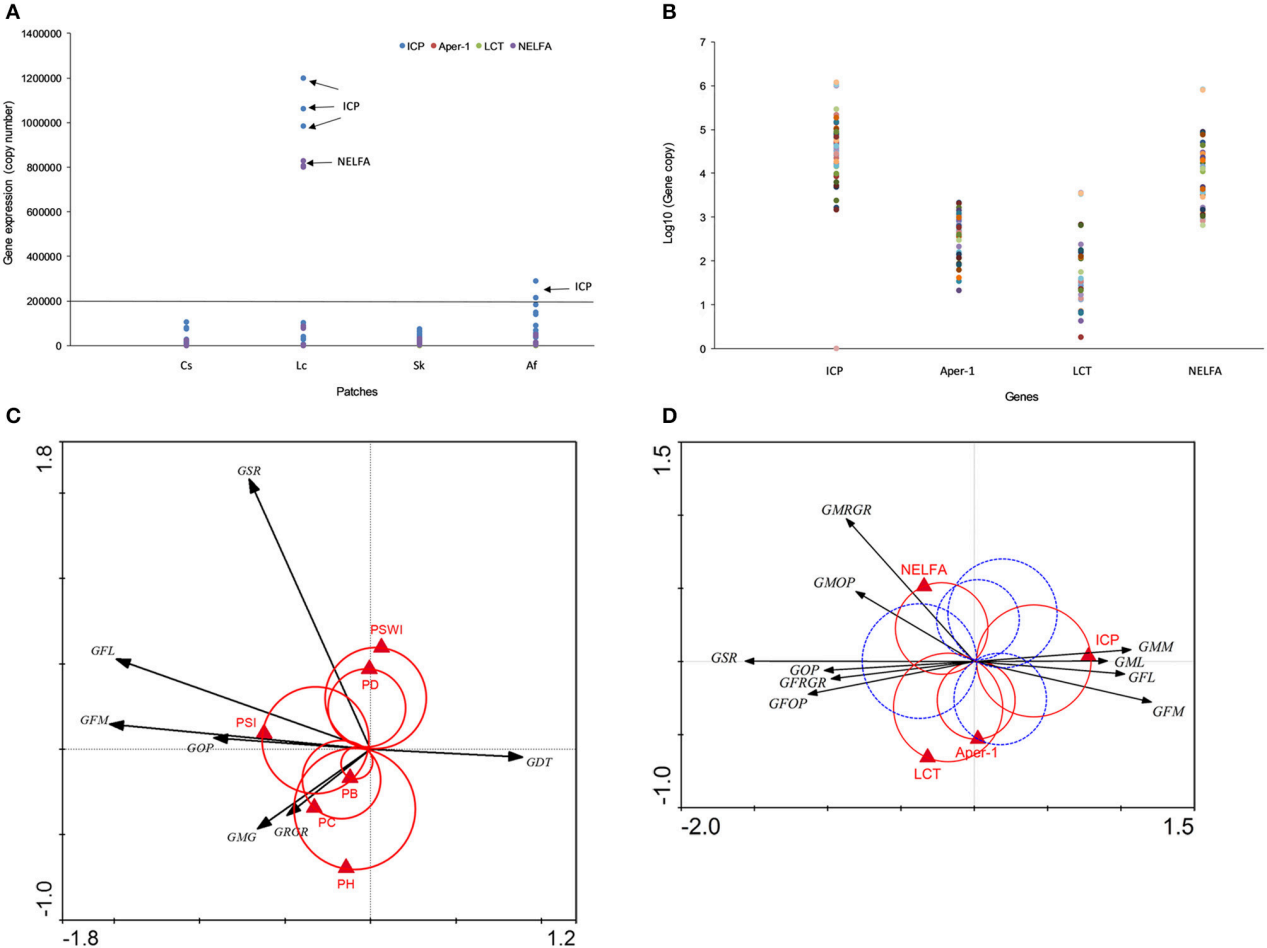
**(A)** Candidate gene expression across four populations detected by qPCR. **(B)** Four gene expression profiles are given. Different colored dots indicate sample individuals, sample size, *N*=48. **(C)** The interaction between grasshopper performance and plant community. The black arrows indicate grasshopper performance, the red Van Dobben circles with the red triangles indicate plant community structure variables and their effects. **(D)** The interaction between grasshopper performance and genes. Black arrows indicate grasshopper performance and the triangles indicate specific genes. The grasshopper performance involved in one of the two color circles predicted to change their value with the changing value of that particular gene. Those preferring higher values of the performance variables are enclosed by the positive circle (red). Those with preference for low values of the corresponding performance variables are enclosed by the negative (mirror) circle (blue dash). GFM, grasshopper female mass; GRGR, grasshopper relative growth rate; GMG, grasshoppers' mass gain; GFL, grasshopper female length; GDT, grasshopper development time; GSR, grasshopper survival rate; GOP, grasshopper overall performance; PSWI, plant Shannon-Wiener index; SI, plant selective index; PH, plant height; PD, plant density; PB, plant biomass; PC, plant coverage; Aper1, peritrophin-1; ICP, insect cuticle protein; NELFA, negative elongation factor A; LCT, lactase-phlorizin hydrolase. All genes were at *q*<0.005, |log2.Fold_change|>1.

In order to verify the roles of above candidate genes in grasshopper performance, we cloned their DNA fragments and quantified gene expressions by quantitative real-time polymerase chain reaction. QPCR data indicated that candidate gene expressions for four treatments were consistent with the FPKM data in transcriptome. ICP (*N* = 48, *P* = 0.0143), Aper-1 (*N* = 48, *P* < 0.0001), NELFA (*N* = 48, *P* = 0.0668), and LCT (*N* = 42, *P* = 0.0003) exhibited significantly different expression levels among grasshopper populations across four patches (Figure 4A; Supplementary Figure 6). Sequences for Mpv17/PMP22 failed, however, possibly due to the random spliced fragments having a lower similarity to Mpv17/PMP22.

Intriguingly, grasshoppers in Lc and Af patches showed exceptionally higher gene expressions in LCT and NELFA (Figures 4A,B). Moreover, LCT and NELFA also presented relatively higher levels of expression than other genes across all populations (Figure 4B), suggesting the potential importance of their roles in PP. To explicitly demonstrate the eco-trans-biological relationship, we further constructed the eco-transcriptomic architecture of plastic traits using PP data, environment data, and gene expression data (qPCR data), as shown in the below section.

### Eco-transcriptomic architecture of phenotypic traits

We demonstrated the eco-transcriptomic architecture of plastic traits here by elucidating how grasshopper performance was affected by patches compositions (Figure 4C) and how grasshoppers' performances were tied to specific gene expression (Figure 4D). Plant preference trait (food plant preference, *PSI, W weight* = 1.7) was the main determinant of overall grasshopper performance, while other environmental variables only affected one or more traits that partially account for overall performance. Overall performance was also determined by survival and growth rates. However, growth rate, survival rate, and body size parameters were not clustered tightly together. Rather, they showed highly divergent directions in response to environmental selections. This decentralizes the effect of environmental variables on overall performance, so that grasshoppers can balance the survival rate and growth. Just as Figure 4C depicts, plant coverage, plant height, and plant biomass both could significantly influence grasshopper body weight increments and relative growth rate but not survival rate, while plant density and plant diversity mainly affected the grasshopper survival rate rather than growth rate. This indicated that vegetation structural properties (plant coverage, height, biomass and density) could only influence grasshopper survival or growth rate that partially affect the overall performance.

Gene-performance plotting showed that the different measures of grasshopper performance were mainly controlled by insect cuticle protein (ICP), negative elongation factor A (NELFA), and lactase-phlorizin hydrolase (LCT) (Figure 4D). However, insect cuticle protein (ICP), located at the horizontal axis, appears to be the core gene that can fully explain the variations in performances. That is, high expression values of ICP could increase grasshopper body size (female length and mass, male length and mass), but decrease grasshopper survival rate and overall performance (See GSR, GFOP, GMOP, GOP in Figure 4D, red circle). Conversely, low values of ICP have an opposite effect.

Equally important, negative elongation factor A (NELFA), or lactase-phlorizin hydrolase (LCT), functioned in a supplemental role partly responsible for male growth rate and male overall performance. That is, high values of NELFA only significantly increased male growth rate and male overall performance, while high values of LCT only increased female growth rate and overall performance. NELFA and LCT likewise positively correlate (*N* = 42, *r* = 0.976, *P* < 0.0001) and mutually affect grasshopper performance. While high values of NELFA and LCT may act inversely to high values of ICP for overall performance (Figure 4D), their true values of gene expressions positively correlate as well [correlation: *r* (ICP & LCT) = 0.938, *N* = 42, *P* < 0.0001; *r* (ICP & NELFA) = 0.966, *N* = 48, *P* < 0.0001]. This indicates that ICP, NELFA, and LCT genes might be bound up with each other in molecular events, but act differently on grasshopper phenotypic traits. In contrast, Aper-1, located at the vertical axis, has little to do with the performance variables. ICP, NELFA, and LCT appear as the main determinants of grasshopper performance.

## Discussion

Phenotypic plasticity (PP) is a universal feature of life on Earth (West-Eberhard, 2003; Scheiner and DeWitt, 2004; Whitman and Ananthakrishnan, 2009). Most, if not all, species exhibit some form of PP, and this plasticity manifests at all levels of biological organization (biochemically, physiologically, developmentally, morphologically, behaviorally, as well as in terms of life-history, species-interactions, community structure, and evolutionarily) (Reuter et al., 2017). The changes resulting from PP profoundly influence individual survival and fitness, population densities and biogeography, ecological interactions, and evolution (Kokko et al., 2017; Real, 2017). As such, understanding how genes and the environment interact to regulate PP is of fundamental significance for ecology and evolution. However, while PP has been a topic of importance for biologists for more than 60 years, using molecular tools to study it has become only recently available (Whitman and Ananthakrishnan, 2009; Zhou et al., 2012a; Hunt and Hosken, 2014; Zhang et al., 2016; Zhu, 2016; Hales et al., 2017; Liu et al.; Schneider and Meyer, 2017; Takahashi, 2017; Vendrami et al., 2017; Ziv et al., 2017). As such, there is much that we do not know about PP.

In this paper, we conducted an analysis for differential gene expression in response to forced exposure to a variety of naturally occurring habitat types with *O. asiaticus*. By bridging ecological, biological, and transcriptomic relations with measures of organismal performance, we sought to address six fundamental questions about PP: What factors induce PP? How likely are the organisms we tested to exhibit PP? How pervasive is PP in individuals? What mechanisms underlie PP? What is the spatial-geographic component of PP? Is PP positively related to stress?

We examined the above questions by rearing *O. asiaticus* in the field in four different adjacent plant-communities, and then comparing performance and resulting transcriptomes. Our gene-environment association study suggests that: (1) PP can manifest over surprisingly small geographical/spatial scales; (2) PP is highly subject to environment conditions and the effects are pervasive, affecting manifold traits; (3) environmental selection favors adaptive plasticity; and (4) ICP, NELFA and LCT are crucial genes that underpin PP.

### Small habitat differences can induce differential PP across small geographic distances

In our field experiment, we raised grasshoppers in four adjacent, flat grassland sites that differed moderately in various vegetative characteristics, including plant diversity, grass cover and height, vegetation density and biomass, and presence of favored host-plants (Table 1). However, all replicates were similar in terms of grasshopper ages, sex-ratios, and densities, and all replicates lacked predators and competitors. All four sites were within a 200-m diameter, flat “grassland ecosystem,” and all experimental animals were obtained from the same population. This aforementioned approached was intended to eliminate grasshopper social aspects, age, predator- and competitor-threats, and genetic issues from influencing the results. As such, the treatments were similar in slope, edaphic, photoperiod, and macroclimate characteristics.

Nonetheless, the moderate environmental differences among the four treatments (Table 1) induced multiple, significant performance disparities in the grasshoppers (Table 2), undergirded by transcriptional differences (Figures 1–3; Supplementary Tables 5, 6; Supplementary Figure 4). This suggests that, in addition to food plant type, small, local habitat differences, such as plant density, plant cover and biomass, and their accompanying physical differences (sunlight, temperature, humidity, wind, etc.), can serve as cues or factors that induce pervasive PP in relatively short time spans.

This is an important finding, in part, because there are widespread differences of viewpoint in PP studies, some of which assume that PP requires major habitat differences or threats, such as winter vs. summer, low vs. high altitude, or predator/pathogen presence (Whitman and Agrawal, 2009; Whitman and Ananthakrishnan, 2009). Other contrasting evidence, along with niche theory, has supported the assumption that small-scale environmental filtering can induce high PP. Our results imply that substantial PP can occur in response to relatively minor environmental differences, and over small geographic distances. We know that local habitats vary spatially in multiple small ways. Our study suggests that organisms living only meters apart may express PP-based differences. Hence, local populations experience a much finer scale PP, with, perhaps, each individual adjusting its phenotype in relation to its immediate surroundings and own experiences (Whitman et al., 2009).

### Evolution of phenotypic plasticity: habitat structural properties favor adaptive plasticity

Stress plays a major role in two general types of PP: (1) susceptibilities and (2) adaptive (evolved) PP (Whitman and Agrawal, 2009; Whitman and Ananthakrishnan, 2009). Harmful stress factors (poor nutrition, cold, heat, pathogens, toxins, crowding, etc.) disrupt physiological homeostasis in susceptible individuals with manifold interactive changes to the phenotype. Concomitantly, adaptive PP can represent an evolved countermeasure to harmful stress (Whitman and Agrawal, 2009; Whitman et al., 2009). In both cases, various stress factors can directly induce altered gene expression (Whitman and Agrawal, 2009; Enders et al., 2015).

In this study, the degree and direction of PP associated with various stress factors experienced by the grasshoppers, with nutritional stress predominating. *O. asiaticus* expresses strong feeding preferences. Our laboratory feeding trials showed that food preferences in *O. asiaticus* were Sk >Cs >Lc >Af. Speed of development, female growth rate, survival rate, and overall performance in the Af habitat were lower than in other treatments (Table 2). Af treatments contained the smallest ratios of the favored host plant, and thus were presumably the most nutritionally stressful. Hence, grasshopper performance tended to be low in patches with reduced abundance of favored host plants. Furthermore, plant extrinsic functional traits, plant density, and coverage could limit the shelter space and pose a thermal environment for grasshoppers. Likewise, the Lc treatment (having the highest plant coverage and density) showed the lowest female body mass and length (Table 2).

PP can be induced by numerous factors. In our experiment, percent of grass cover, vegetation density, and vegetation biomass influenced PP. Across all four treatments, both female body length and body mass negatively correlated with plant density. Development rate was also slowed by higher plant density and plant biomass (Figure 3C). Hence, a lush plant community (high density and biomass) was unfavorable to the growth and development of this species and produced smaller, lighter grasshoppers. These findings coincide with previous studies that the environmental microstructures—including vegetation structure, fine scale distribution of food plants, and warm spots—can influence the behavior and spatial distribution of grasshopper (Bouaichi et al., 1996; Baythavong, 2011; Vendrami et al., 2017). Inasmuch as grasshoppers tend to be thermomaximizers (Kong et al., 2016), where shade limits solar-heating, thereby decreasing grasshopper body temperatures, this subsequently decreases metabolic rate as well, which is positively related to body temperature. Dense vegetation also produces high humidity, which can benefit grasshopper pathogens.

Grasshopper habitat selection and food selection are the main determinants of grasshopper distribution and outbreak, while studies have indicated that *O. asiaticus* abundance was negatively quadratic correlated with plant coverage, density, and height (Zhang et al., 2013; Hao et al., 2015), and it was often found in sunny sites with ample bare ground (Schmidt and Lilge, 1997; Ingrisch et al., 1998; Zhang et al., 2015). In accordance with the outbreaks of *O. asiaticus* in lower-vegetation, heavy grazed habitats (Cease et al., 2012), our study showed that sparse, bare vegetation habitats produce larger and heavy *O. asiaticus*, whereas dense, high vegetation habitats favor smaller, lighter *O. asiaticus*. Smaller body size, larger hind legs are favorable migratory phenotypes (Cease et al., 2017). This suggests that apart from high-quality diets, the habitat vegetation structural properties have been playing an important role in regulating locust/grasshopper migration/plague in Inner Mongolia grassland.

Furthermore, In the course of habitat environmental selection during fourth instar to adult, grasshopper performance exhibited flexible plasticity that counter-acts stress which can be reflected by the trade-offs between phenotypic traits. This represents the evolution of phenotypic plasticity that benefit the final fitness of grasshopper (overall performance). According to our results, we concluded two evolutionary significances on phenotypic plasticity. (i). Ecological trade-offs between survival and growth that confer high fitness when population sizes (or survival rates are higher) are high and resources are abundant (growth rate is higher). Nevertheless, some factors might decrease growth rate, but benefit survival, or in reverse, which counteracts the decrease of overall performance (Figure 4C). (ii). Physiological trade-offs between body size and overall performance that confer high fitness when the body size (body mass and length) is small regardless of population size and survival rate (Figure 4D). Higher overall performance is at the cost of reducing body size.

### What is the molecular basis of phenotypic plasticity?

Each of the four treatments exhibited both similar and divergent gene expression. The Lc habitat had the highest expression rate, follow by Af, Cs, and Sk (Supplementary Figure 4), but Af had the highest number of differently expressed genes (Figure 1B). These differences relate to variation in grasshopper performance. Systematic characterization of expression patterns associated with specific biological process, and in response to specific physiological perturbations, provides a framework for interpreting the biological significance of the expression patterns observed in each habitat. Indeed, we identified clusters of physiologically relevant co-expressed genes related to specific biological features among the samples.

Gene expression is a complex process that bridges the phenotype and fitness. An ordination triplot of grasshopper-genes-plant communities (Figure 3C) showed that grasshopper performances were correlated to specific gene expression and plant community structure. Five genes, differently expressed among the four treatments, were filtered as PP-related genes: insect cuticle protein (ICP), peritrophin-1, lactase-phlorizin hydrolase (LCT), Mpv17/PMP22, and negative elongation factor A (NELFA) (Table 3). A *T*-value biplot diagram of grasshopper-genes expression (Figure 4D) showed that ICP, NELFA, and LCT are responsible for much grasshopper PP, revealing the underlying unique gene regulation of PP stimulated by specific habitat factors.

Differences in phenotypic variability are primarily the result of genetic interactions (Ziv et al., 2017). Importantly, we found that some traits have a dual relationship between quantitative gene expression and environments in contrast to others that are unique and show a gene-by-environment interaction. Ecologically, grasshoppers had divergent phenotypic traits can respond to different environmental variations (Figure 4C) and thus adapt plasticity to environmental stresses. Molecularly, body size (female length, mass; male length, mass) and performance differed inversely and were regulated mainly by ICP and only partially by NELFA and LCT (Figure 4D). This indicates that body size and performance compensate one another, suggesting a cost-balance between body size and performance physiologically. This suggests why and how grasshoppers ecologically and molecularly can develop the ability to adapt to environmental variation through PP.

**Figure 5:**
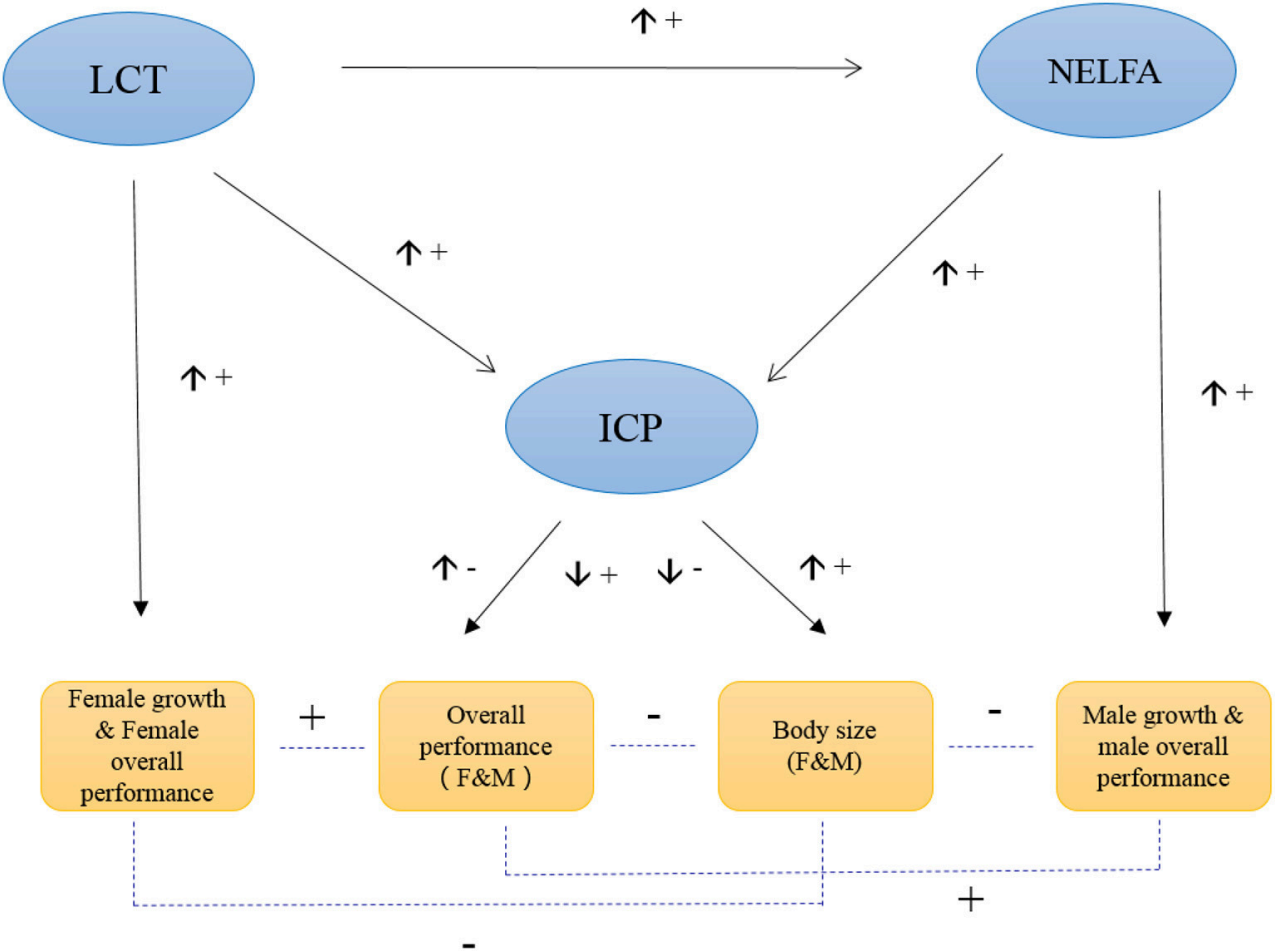
Hypothesized model of the molecular control of grasshopper phenotypic plasticity. The up arrow (↑) beside the connecting line indicates the expression of the upstream gene is increasing, while down arrow (↓) indicates the expression of upstream gene is decreasing. “+” indicates the positive effect, while “−” indicates the negative effect.

In general, ICP plays a central role in regulating grasshopper PP. Its expression controls grasshopper fitness by balancing the plasticity of body size against overall performance. That is, its high expression value increases grasshopper body size while decreasing overall performance. Intriguingly, NELFA and LCT play sex-specific roles in PP, and function as supplemental genes responsible for male performance and female performance, respectively (Figure 5). In general, ICP, NELFA, and LCT act interactively and complementarily to determine the phenotypic plasticity of grasshopper under environmental selection. But these aforementioned hypotheses remain to be tested, beyond the scope of this paper, in future studies if or when RNAi strategies (or other functional genomic tools) may become available for this species.

## Conclusions

Under a framework of phenotypic plasticity, individuals exhibit different plastic traits under different habitats; our results are in keeping with this hypothesis. But beyond that, our study suggests that organisms may be more prone to PP over a finer environmental scale than previously thought. Our study also contributes to the on-going elucidation of molecular mechanisms associated with PP. In this paper, we highlighted transcription as a primary mechanism for facilitating PP. However, numerous other mechanisms also act to produce PP, including direct effects of environment on translation, enzyme co-factors, hormones, or the nervous system (Kang et al., 2004; Akman and Whitman, 2008; Aubin-Horth and Renn, 2009; Whitman and Agrawal, 2009; Ma et al., 2011; Harrelson and Valentino, 2013; Tanaka et al., 2016; Chevin and Hoffmann, 2017; Hales et al., 2017; Lea, 2017; Mank, 2017; Reuter et al., 2017; Ziv et al., 2017). Future studies, using proteomic or metabolomics tools, may help to better define other mechanisms, beyond just transcriptional differences, involved in PP. Most likely during phenotypic change, multiple molecular mechanisms (e.g., transcriptomic, proteomic, and metabolomic) act simultaneously and in sequence, with manifold interactive effects (Sun et al., 2011). A systems-scale analysis may reveal more complex interactions beyond what was observed transcriptomically (Sun et al., 2011). Additionally, we fully recognize that the timing of when the samples collected gives us only a snapshot in time of the overall transcriptional response and hence if the samples were taken at a different time, we may have observed other underlying factors associated with PP. Finally, reverse genetic strategies, e.g., RNAi, will be necessary to fully test the direct role of many of these factors, observed in these analyses, in PP (Pittendrigh et al., 2015).

Constructing an eco-transcriptomic architecture of plastic traits is a relatively new approach and has some unique advantages over traditional approaches for identifying a trait's genetic architecture (Hunt and Hosken, 2014). In our study, we demonstrated the eco-transcriptomic architecture of PP by examining how community composition acts on the genome to produce PP in *O. asiaticus*. We also documented an association between evolution, PP, and fitness. From an ecological perspective, grasshopper survival rate and growth decentralize the environmental effects so as to minimize the environmental impacts on overall performance. From a physiological perspective, grasshopper body size and overall performance would act compensatorily so that grasshoppers can regulate the performance (fitness) by the expression of plastic body traits according to the environmental selection. Grasshoppers that were most stressed, also expressed the highest PP and lowest fitness. Thus, the correlation linking higher PP to lower fitness suggests a physiological cost to plasticity.

In sum, our study emphasized the eco-biological-molecular aspect of PP and its evolution in relation to pest management, while other molecular or biological approaches can't reach. There is still a long way to go to achieve an adequate understanding of the molecular mechanisms underlying PP, however, this study provides an important step toward that goal.

## Materials and methods

### Study site and organisms

Experiments were conducted at the Scientific Observation and Experimental Station of Pests (SOESP), in Xilingol Rangeland, Ministry of Agriculture, Xilin Gol League, Inner Mongolia, China (43°95′N, 116°01′E). This region consists primarily of grassland, with considerable local variation in plant height, density, and cover, plant species diversity, and grass-species dominance (Shen et al., 2015). We used the grasshopper *Oedaleus asiaticus* Bey-Bienko, 1941 (Orthoptera: Acrididae) as our study organism.

This grassland inhabitant ranges from the north Asian steppe to Central-Asia and China, and is particularly abundant throughout the Inner Mongolia steppe, the largest grassland in China (Li et al., 1990). It undergoes periodic population outbreaks that result in massive numbers of grasshoppers that destroy rangeland vegetation and cause economic and social harm (Zhou et al., 2012b; Zhang et al., 2015). The species is moderately polyphagous, but favors specific food plants (Cease et al., 2012). While thermophilic, and preferring to stay at sunny sites with ample bare ground (Schmidt and Lilge, 1997; Ingrisch et al., 1998; Zhang et al., 2015), it will also shuttle through other patches if community compositions are changed (Zhang et al., 2015). Eggs were laid in the ground in fall, allowed to overwinter, and then hatched in early June. The insects underwent five instars, molted to adults in late July, and mated and oviposited from August to September (Hao and Kang, 2004).

### Field study

To examine how habitat differences influenced animal PP, we reared *O. asiaticus* grasshoppers in four different grassland types (= four treatments) dominated, respectively, by *S. krylovii* Roshev., *L. chinensis* (Trin.) Tzvel., *C. squarrosa* (Trin.) Keng., or *A. frigida* Willd. We abbreviate these treatment areas as Sk, Cs, Lc, and Af, respectively.

Sk, Cs, and Lc are grasses (fam. Poaceae); *A. frigida* is in the family Asteraceae. Each of these plant community types are common in the area, with each plant species occupying 90% of their patches plant composition.

On 20 June 2014, we collected ~2,000 second instar *O. asiaticus* by sweep net from locust infested areas in West Ujimqin—90 km from the experimental station, and located in east Xilin Gol, an area with low, sparse vegetation mixed with Sk, Cs, Lc, Af. Collected specimens were placed into four outdoor rearing cages (2 × 1.5 × 2 m) at SOESP until the third instar, at which time when they were randomly assigned to one of the four treatment groups.

Each treatment included five replicates, consisting of 10 males and 10 female nymphs in a communal cage, or fifty males and fifty females/treatment. Treatment cages were 1 × 1 × 1 m and constructed of 1-mm cloth mesh over an iron frame. We selected our mesh coverings for high ventilation and photopermeability, so as to keep physical conditions inside and outside the cage as similar as possible. The cages were open on the bottom, and were placed on the ground outdoors, over the natural vegetation, which allowed the grasshoppers to feed at will. Competing insects, such as other insect herbivores, and natural enemies, such as spiders, were removed from inside the field cages before adding the grasshopper nymphs. The experiment continued for 27 days, during which the grasshoppers fed on the wild, growing plants inside their cages, and developed from third instar to adults.

All treatments were conducted within a 200-m diameter range, providing a similar macroclimate for all replicates. Moreover, in 2015, we repeated the experiment by enlarging the replicates to 20 cages with 400 individuals per patch. To examine the coincidence of candidate gene expression with RNA-Seq, we collected the samples and identified their gene expressions.

Data were collected on: (1) the number of survivors in each of the 20 cages, every 2 days until the grasshoppers reached adulthood; (2) the development time from Day 1 (the start of the experiment, when all insects were already third instar) to the adult molt, which occurred mostly between Days 22 and 24; and (3) female body length and fresh (wet) mass of third instars on Day 1 and 3- to 5-day-old adults on Day 27, using Vernier calipers and an analytical balance on 10 males and 10 females (four randomly selected from each replicate) each of the four treatments; and (4) plant species, % coverage (% of ground covered by vegetation), height, density (number of individual plants/m^2^), plant diversity (Plant Simpson's diversity index and Plant Shannon-Weiner index) and above-ground biomass (dry weight) from the beginning, middle, and end of the 27-day experiment period for each of the four plant-habitat types.

### Food preference test

In a separate laboratory experiment, we determined *O. asiaticus* feeding preferences for the four main plants in the study (*S. krylovii, L. chinensis, C. squarrosa*, and *A. frigida*). Trials were conducted in five 6-L volume plastic boxes for each sex; two adult females or two adult males/box maintained in environmental chambers at 30°C ± 1°C and 16:8 L: D photoperiod. Insects were approximately 10 d old as adults at the start of the experiment, were initially starved for 24 h, and then tested for 6 d. Every 2 d at dawn, fresh plant material was obtained from the nearby field and brought to the lab. There, 2 g (±0.01 g) lengths of leaf were cut from each of the four plants. The base of each 2 g sample was inserted into a single water-filled tube. Each cage was given four such tubes every other day. The grasshoppers were free to choose among the four plant species for 48 h. Each day, dead grasshoppers were replaced with new ones. Every 2 d, old leaves were replaced and the amount of plant consumed by the grasshoppers was quantified. The remaining portions of uneaten leaves were collected after 48 h, dried for 24 h at 80°C to constant weight, and weighed. Dry mass eaten (*E*) was calculated by subtracting the dry mass of the uneaten leaves (*U*) from an estimate of the original dry mass (*O*) for a given sample. We used similar methods to prepare 10 replicates of control leaf samples, except that the control cages lacked grasshoppers. Comparisons of wet and dry masses of control vs. treatment leaf samples allowed us to calculate both wet and dry grasshopper consumption and total water intake of each of the four plants offered. From this we calculated feeding preferences. In total, we tested five male and five female cages, each for 6 d, divided into three consecutive 2 d periods.

In the Results section, we report grasshopper feeding preferences as a Selective Index (*SI*) for females, calculated as: *SI* = D/P, where *D* is the percentage dry mass of a food item in the diet, and *P* is the percentage dry mass of the same food plant species supplied (Van Dyne and Heady, 1965).

### Statistical analysis

Differences in habitat conditions (plant diversity, density, coverage, height, biomass), selective index (*SI*) for females, among dominant plants, and grasshopper performance [relative growth rates and survival, female body weight, female body length, female overall performance (= relative growth rate × survival rate), the data was 100-fold and log_10_ transformed] in the four habitat treatments were compared via one-way analysis of variance (ANOVA) followed by Tukey's Studentized Range (HSD) at *P* < 0.05 using SAS 8.0 software (SAS Institute, Cary, NC, USA). Correlation among environmental variables, performances, and genes expression were tested using Pearson Correlation Coefficients. Canonical correspondence analysis (CCA) was used to express factors affecting grasshopper performance and to illustrate relationships between grasshopper species, plant community structure, and specific gene expression. We used experimental plots as samples, the grasshopper performance variables as species data, patch environmental variables as environmental data, and gene expression level as covariable data. “Don't transform with Monte-Carlo permutation test” was used (number of permutations 999, full model) to indicate the main factors and correlation. CCA analyses were completed using CANOCO 4.5. CCA plot (Figure 3C) and *T*-value plots (Figures 4C,D) were made using CanoDraw 4.5. Other figures were made by Origin 8.0.

### Samples for transcription analysis

We obtained samples for transcriptional analysis by randomly selecting one 5 d old adult female from each replicate-cage, for 20 total (5 replicates/treatment × 4 treatments = 20 samples). Females were frozen in liquid nitrogen (Air Liquide, Voyageur 12) and preserved at −80°C until RNA analysis. We prepared sequencing and cDNA libraries from the treated four samples. Five individuals were combined for each treatment were used for sequencing; cDNA libraries (Cs, Lc, Sk, and Af) were prepared using the reference transcriptome preparation method (Tariq et al., 2011).

### Preparing the total RNA and cDNA libraries and illumina sequencing

We combined and homogenized the same amount of head, thorax, abdomen, legs, and ovaries dissected from five individuals (one individual per cage) in the same treatment (= habitat). The total RNA of each treatment was extracted using the TRIzol reagent (Invitrogen, California, USA) following the manufacturer's instructions (https://tools.thermofisher.com/content/sfs/manuals/trizol_reagent.pdf). Illumina sequencing was conducted by Novogene Corporation.

### Reference transcriptome assembly and annotation

Data were combined for each treatment for reference assembly. Supplementary Table 7 lists the software and parameters used for non-reference transcriptome assembly and analysis. BLAST searches against the non-redundant (NR) and nucleotide sequence (NT) databases NCBI, SWISS-PROT, KEGG, and KOG were performed with an *e*-value cut-off at 1e-5. Gene Ontology terms were assigned using Blast2GOv2.5 (Götz et al., 2008) by searching the NR database.

### Gene expression levels

Clean reads (extracted by the screening sequenced data process used in the preparation of reference transcriptome) for each sample were mapped onto the reference transcriptome. In the mapping process, RSEM software was used following the manufacturer's instructions (Li and Dewey, 2011). Mapping results from RSEM were calculated to generate the read count for each gene and converted to FPKM (fragments per kilobase million) using the estimation method (Mortazavi et al., 2008). An FPKM density distribution was generated to verify the expression profile of each sample.

### Analyzing the differentially expressed genes

To detect the FPKM distribution, the gene FPKM density distributions were compared among treatments. Using screening threshold at p. adj < 0.05, DESeq was used to analyze the read count data and to identify differentially expressed genes under different patches (Mortazavi et al., 2008). We used *D*_gene_ = log_2_(a/b) (a is the FPKM of sample a, and b is the FPKM of sample b) to do pairwise comparisons between treatments to find the difference in gene expression. When the value of *D*_gene_ is larger than 2, or less than−2, it implies that gene expression between the two samples is significantly different or they do not have any difference. The total number of differentially expressed candidate genes was calculated by adding up the differentially expressed genes across all comparisons but avoiding overlap.

### GO enrichment analysis

Gene Ontology (GO) enrichment analysis of the differentially expressed genes (DEGs) was implemented with GOseqR using a Wallenius non-central hypergeometric distribution (Young et al., 2010), which can adjust for gene length bias in DEGs.

### KEGG analysis of the significant enrichment of differentially expressed genes

Using the KEG database, we performed a pathway enrichment analysis to identify the main biochemical pathways and signal transduction pathways involved in differentially expressed genes. Downstream products of the differentially expressed genes in various pathways were investigated as well to identify the substrate that affects patches responses.

### Quantitative validation of candidate gene expressions by real-time PCR

#### Grasshopper sampling and DNA preparation

In 2015, we repeated the above experiment by increasing the biological replicate from 5 to 20 per patch. Due to the larger sample size, we randomly selected five adult females and five adult males from each cage, with every treatment (patch) comprising four replicates, repeated three times for PCR detection. We ground the samples from each replicate for RNA extraction using RNAprep pure Tissue Kit (TIANGEN Biotech Co., Ltd., China), then reversed the RNA samples into cDNA using reverse transcriptase (TIANGEN Biotech Co., Ltd., China).

#### The standard curve construction

First we constructed the standard carve for each gene. We extracted the RNA from sample grasshoppers, reversed it into DNA according to the commonly used protocols, and then used a SYBR Premix Ex Taq II kit (TaKaRa) to conduct the PCR in iQ5 Sequence Detection System (Applied Biosystems, Bio-Rad Company, American). According to manufacturer's instructions, the real-time PCR protocol was performed in a final volume of 25μL. Each tube contained: 2μL sample DNA (10 ng); 12.5μL SYBR Premix Ex Taq II; 1μL (20μM) of forward primer; 1μL (20μM) of reverse primer. Finally, we added ddH_2_O to provide a volume of 25μL. The PCR protocol consisted of one step at 95°C for 30 s, followed by 45 cycles at 94°C for 20 s, the respective optimized annealing temperature (as described above) for 20 s, 72°C for 20 s and a final cycle of 72°C for 5 min. No-template controls (NTC) were conducted to detect possible sample contamination. To ensure that only the desired product was amplified, a dissociation curve was produced for each reaction to monitor fluorescence continuously. All real-time PCR were performed in a room dedicated to the quantification to avoid contamination and conducted by a single person. The primer of the four genes listed in the supplementary material (Supplementary Table 8).

We use β-actin as the control (reference) gene for quantitation. The standard curve was generated using DNA from the purified plasmid isolated as the standard. Six concentration gradients (10^3^,10^4^, 10^5^, 10^6^, 10^7^, 10^8^) were performed. We determined concentration (μg/μL) by spectrophotometric measurement (UV-visible Spectrophotometer, UV-2550, Shimadzu) and calculated the numbers of target DNA sequence copies as the number of target DNA transcripts: [DNA mass (μg)/DNA molar mass]×6.023×10^23^. The number of transcripts was calculated for 10 ng, which the volume used as the template in each real-time PCR assay. Through tenfold serial dilutions of the transcripts (dilution ratio 10^2^–10^8^), standard curve equation for the four genes between *Ct-*value and the copied amount of DNA were produced, respectively. Each point on the standard curve was assayed in triplicate. From the standard curve equation, we could determine the copy number of DNA (in log_10_form) for each species according to the value of *Ct*. Real-time PCR amplification efficiencies (*E*) were calculated from slope values of standard curves using the formula −1+10^(−1slope−1)^.

The copy numbers of each gene detected from each treatment were used to model the architecture of phenotypic traits.

## Data accessibility

The transcriptome data of *O. asiaticus* females was submitted to SRA database in NCBI and have been released (ID: SRP059063).

## Author contributions

XQ and ZZ designed the experiments. XQ performed the experiments. XQ analyzed the data. KH and JM provided technical support. XQ, DW wrote the manuscript. XH, XT, GC, GW, XN, BP, and MA provided the improved suggestions and comments. All authors reviewed and considered the manuscript.

## Ethics statement

All experimental protocols and animal studies were approved by the Institute of Plant Protection, Chinese Academy of Agricultural Sciences. We confirm that all experiments were carried out in accordance with the relevant guidelines and regulations.

### Conflict of interest statement

The authors declare that the research was conducted in the absence of any commercial or financial relationships that could be construed as a potential conflict of interest.
