# Supplementary material for: Molecular Ecological Basis of Grasshopper (Oedaleus asiaticus) Phenotypic Plasticity under Environmental Selection
Source: Front Physiol. 2017 Oct 10;8:770. doi: 10.3389/fphys.2017.00770 (PMC5641302; doi:10.3389/fphys.2017.00770)
Supplement: Supplementary file 1 [file SupplementaryFiguresandTables.pdf]

Supplementary Information

Molecular Ecological Basis of Grasshopper (*Oedaleus asiaticus*) Phenotypic Plasticity under Environmental Selection

Xinghu Qin[1,2,3], Kun Hao[1], Jingchuan Ma[1,3], Xunbing Huang[1,3], Xiongbing Tu[1,3], Md. Panna Ali[4], Barry R. Pittendrigh[5,] Guangchun Cao[1,3], Guangjun Wang[1,3], Xiangqun Nong[1,3,] Douglas W. Whitman[6] & Zehua Zhang[1,3**]

[1] State Key Laboratory for Biology of Plant Diseases and Insect Pests, Institute of Plant Protection, Chinese Academy of Agricultural Sciences, Beijing, 100193, P.R. China
[2] School of Biology, University of St Andrews, East Sands, St Andrews, KY16 8LB, Scotland, UK
[3] Scientific Observation and Experimental Station of Pests in Xilingol Rangeland, Ministry of Agriculture, Institute of Plant Protection, Chinese Academy of Agricultural Sciences, Xilinhot, 026000, P.R. China
[4] Entomology Division, Bangladesh Rice Research Institute (BRRI), Gazipur, 1701, Bangladesh
[5] Department of Entomology, Michigan State University, East Lansing, MI 48910, USA
[6] School of Biological Sciences, Illinois State University, Normal, Illinois, 61790, USA

List of Supplementary Information

Supplementary Figure 1. A pie chart of species classification of the top BLAST hits

Supplementary Figure 2. KOG Classification of the annotated genes.

Supplementary Figure 3. KEGG classification.

Supplementary Figure 4. FPKM density distributions of the samples.

Supplementary Figure 5. Specific genes expression patterns.

Supplementary Figure 6. Gene difference analysis across patches detected by qPCR.

Supplementary Table 1. Data output quality for the examined samples.

Supplementary Table 2. Length distribution for the assembled sequences.

Supplementary Table 3. Frequency distribution for the assembled sequences.

Supplementary Table 4. Unigene annotation in the databases.

Supplementary Table 5. Statistics of pathway enrichment between treatments.

Supplementary Table 6. GO enrichment.

Supplementary Table 7. The software and parameters for Non-reference transcriptome assembly and analysis.

Supplementary Table 8. Designed sequences of real-time PCR primers for the candidate genes.

Supplementary Figures

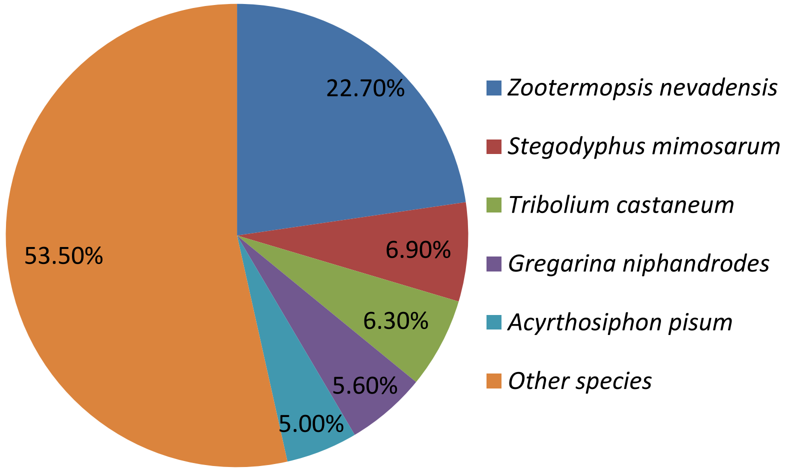

Supplementary Figure 1. A pie chart of species classification of the top BLAST hits.

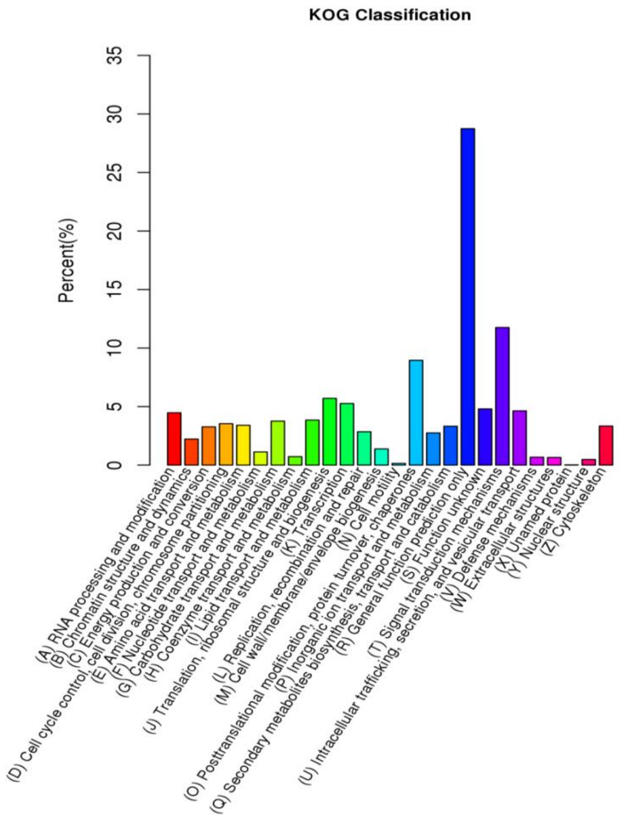

Supplementary Figure 2. KOG Classification of the annotated genes. The genes were classified into 26 groups, horizontal axis is group name of KOG, vertical axis is the percentage of the annotated genes in this terms.

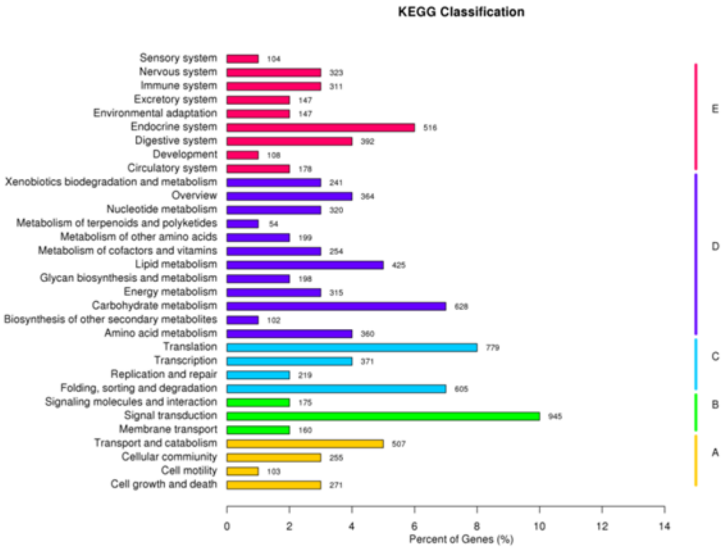

Supplementary Figure 3. KEGG classification. Horizontal axis is percent of genes (%), vertical axis is group name of KEGG. The numbers in the right of bar chart are the number of genes annotated to the classification.

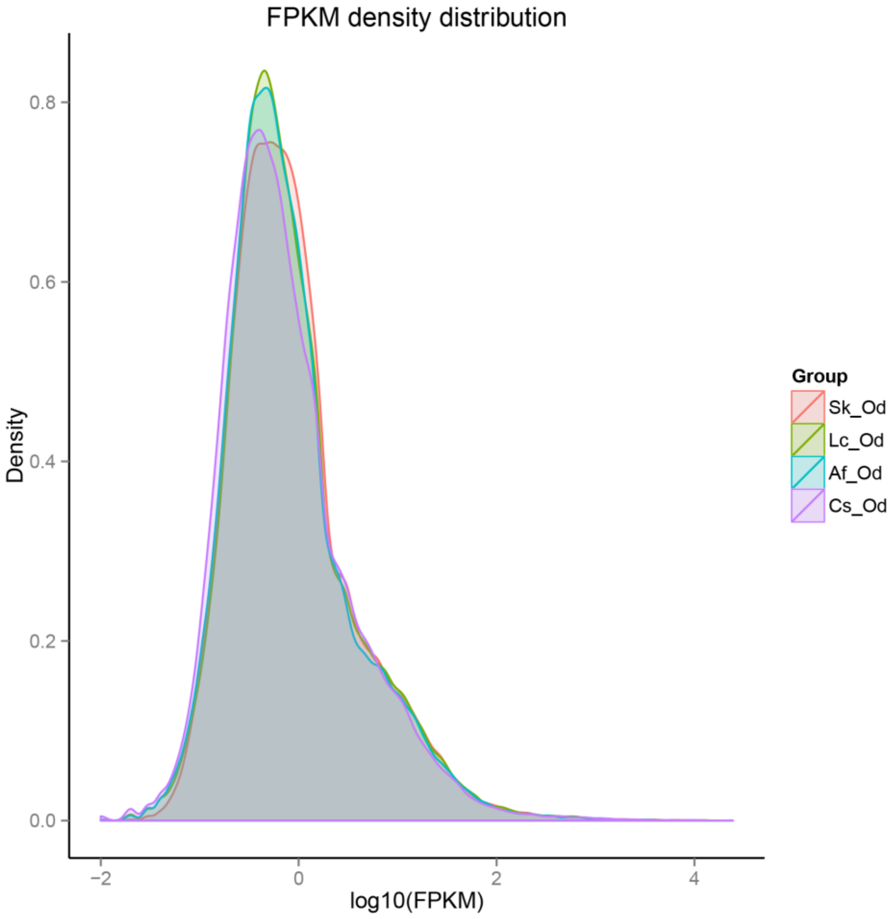

Supplementary Figure 4. FPKM density distributions of the samples. The X-axis indicates log10 (FPKM), and the Y-axis indicates relative density of log10 (FPKM).

A

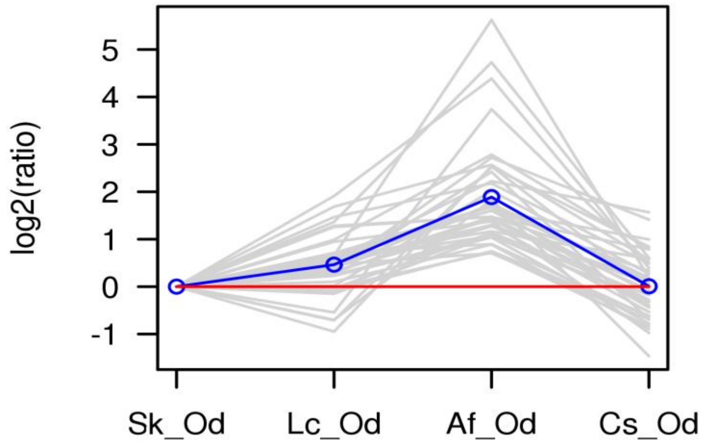

B

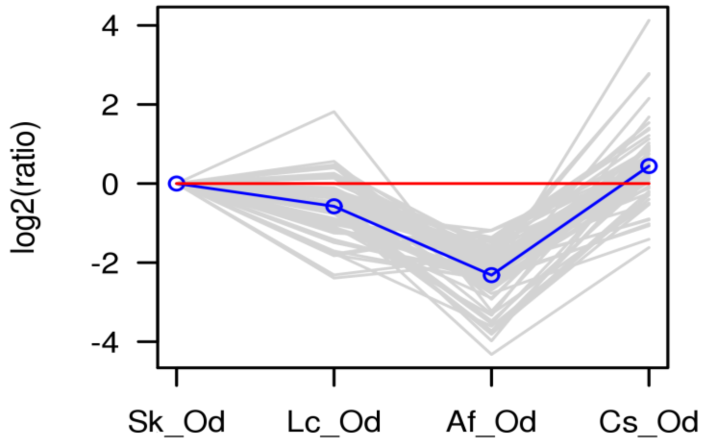

Supplementary Figure 5. Specific genes expression patterns (A) The gene expression pattern 1 (relative gene expression increased with overall performance decreased, 39 genes). (B) The gene expression pattern 2 (relative gene expression decreased with the allover performance decreased, 78 genes). The gray line indicates relative expression [gene log2 (ratios)] in cluster group under different treatments, the blue line indicates the average value of relative expression of all genes. The X-axis indicates treatments, and the Y-axis indicates relative gene expression [log2 (ratios)]. The Sk is set as zero.

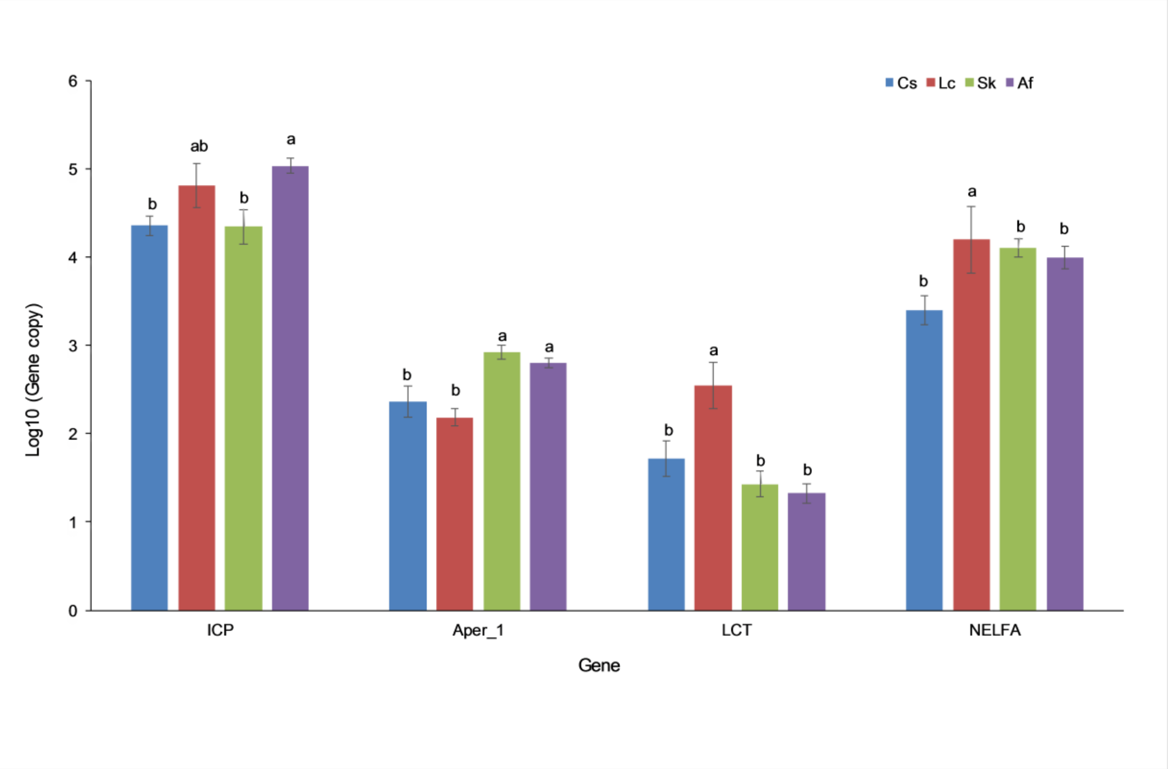

Supplementary Figure 6. Gene difference analysis across patches detected by qPCR. Different lowercases indicate significant difference in their values at $P < 0.05$. Multiple comparison analysis was performed using Tukey's Studentized Range (HSD).

Supplementary Tables

Table 1. Data output quality for the examined samples

Sample	Raw Reads	Clean reads	Clean bases	Error (%)	Q20 (%)	Q30 (%)	GC (%)
Sk_Od_1	45,950,081	45,040,199	5.63G	0.03	97.41	94.71	44.58
Sk_Od_2	45,950,081	45,040,199	5.63G	0.03	95.77	91.99	44.55
Lc_Od_1	43,720,197	42,678,822	5.33G	0.03	97.26	94.4	45.58
Lc_Od_2	43,720,197	42,678,822	5.33G	0.03	95.5	91.48	45.56
Af_Od_1	43,222,878	41,919,760	5.24G	0.03	97.38	94.63	44.35
Af_Od_2	43,222,878	41,919,760	5.24G	0.03	95.57	91.59	44.32
Cs_Od_1	42,614,587	41,669,258	5.21G	0.03	97.24	94.36	45.92
Cs_Od_2	42,614,587	41,669,258	5.21G	0.03	95.43	91.35	45.96

Sample: Sample Name; 1 indicate reads that outputted from left, 2 indicates reads that outputted from right. The total clean reads of the sample was the sum of reads 1 and reads 2. Q20, Q30: the percentage of bases for which the Phred value is greater than 20 or 30; GC content: the percentage of the combined G and C accounts for the total base number.

Table 2. Length distribution for the assembled sequences

	Min Length	Mean Length	Median Length	Max Length	N50	N90	Total Nucleotides
Transcripts	201	855	358	49,475	1,900	285	152,789,985
Unigenes	201	692	325	49,475	1,313	255	100,203,225

N50: the length of the transcript for which the accumulated value is greater than 50% of the total length. N90: the length of the transcript for which the accumulated value is greater than 90% of the total length.

Table 3. Frequency distribution for the assembled sequences

Transcript length interval	200-500bp	500-1kbp	1k-2kbp	>2kbp	Total
Number of transcripts	112,987	28,120	18,304	19,300	178,711
Number of unigenes	100,376	21,656	12,171	10,680	144,883

Table 4. Unigene annotation in the databases

Database	Number of unigenes	Percentage (%)
NCBI non-redundant protein sequences	33,604	23.19
NCBI nucleotide sequences	4,481	3.09
KEGG orthology	9,268	6.39
SwissProt	18,708	12.91
PFAM protein Family	29,232	20.17
Gene Ontology	29,675	20.48
Clusters of orthologous groups of proteins	13,958	9.63
All Databases	2,252	1.55
At least one Database	43,939	30.32
Total Unigenes	144,883	100

Table 5. Statistics of pathway enrichment between treatments

Term	Sample number	qvalue	Gene name
Af vs Lc down			
Amino sugar and nucleotide sugar metabolism	7	0.002205	E3.2.1.14, UGDH, ugd E3.2.1.14, CHS1, glmS, GFPT, UGP2, galU, galF, UAP1
Af vs Sk down			
Galactose metabolism	9	5.12E-05	LCT, malZ, LCT
Amino sugar and nucleotide sugar metabolism	8	0.000199	UGDH, ugd, E3.2.1.14, glmS, GFPT, CHS1
Starch and sucrose metabolism	9	0.001494	E3.2.1.4, malZ, UGDH, ugd, K01176, E3.2.1.28, treA, treF, E3.2.1.21
Af vs Cs down			
Protein processing in endoplasmic reticulum	10	0.001177	CRYAB, htpG, HSP90A, HSPA1_8, CRYAB, HSPA5, BIP, OST1, RPN1, K07151
Cs vs Lc up			
Protein processing in endoplasmic reticulum	9	0.000964	SWP1, RPN2, PDIA3, GRP58, K07151, TRAM1, PDIA1, P4HB, ERP29, HSP90B, TRA1, WBP1, OST1, RPN1
N-Glycan biosynthesis	5	0.001487	K07151, SWP1, RPN2, ALG5,WBP1, OST1, RPN1
Fatty acid metabolism	6	0.00402	FASN, ELOVL6, SCD, desC
Various types of N-glycan biosynthesis	4	0.00402	K07151, SWP1, RPN2, WBP1, OST1, RPN1
Biosynthesis of unsaturated fatty acids	3	0.032894	SCD, desC, ELOVL6
Cs vs Sk up			
Fatty acid elongation	4	0.007417	ELOVL7, ELOVL6
Protein processing in endoplasmic reticulum	8	0.008227	htpG, HSP90A, SWP1, RPN2, SSR4, PDIA3, GRP58, HSPA5, BIP, HSPA1_8, K07151
Fatty acid metabolism	6	0.008227	SCD, desC, FASN, ELOVL6
Sk vs Lc up			
Galactose metabolism	9	6.81E-06	malZ, LCT
Sk vs Cs up			
Galactose metabolism	15	1.25E-08	LCT, malZ, GLA
Amino sugar and nucleotide sugar metabolism	11	3.50E-05	UGDH, ugd, E3.2.1.14, glmS, GFPT, CHS1, HEXA_B, UAP1
Starch and sucrose metabolism	10	0.030312	UGDH, ugd, malZ, K01176, E2.4.1.1, glgP, PYG, E3.2.1.21, uidA, GUSB
Lysosome	9	0.036999	CTSC, ATPeV0A, ATP6N, SLC17A5, uidA, GUSB, NAGLU, HEXA_B, ATPeV0C, ATP6L, GLA

All genes were at qvalue < 0.05, |log2.Fold_change| >1.

Table 6. GO enrichment

Af vs Lc				
GO accession	Description	Term type	Corrected pValue	DEG item
GO:0006030	chitin metabolic process	biological process	6.33E-18	22
GO:1901071	glucosamine-containing compound metabolic process	biological process	6.33E-18	22
GO:0008061	chitin binding	molecular function	8.3E-17	21
GO:0006040	amino sugar metabolic process	biological process	2.75E-16	22
GO:0097367	carbohydrate derivative binding	molecular function	4.55E-15	21
GO:0006022	aminoglycan metabolic process	biological process	5.8E-15	23
GO:0042302	structural constituent of cuticle	molecular function	5.48E-12	15
GO:0005576	extracellular region	cellular component	0.001121	38
GO:0016798	hydrolase activity, acting on glycosyl bonds	molecular function	0.009256	15
GO:0004553	hydrolase activity, hydrolyzing O-glycosyl compounds	molecular function	0.009662	14
GO:1901135	carbohydrate derivative metabolic process	biological process	0.040413	30
Af vs Sk				
GO:0006030	chitin metabolic process	biological process	1.83E-24	27
GO:1901071	glucosamine-containing compound metabolic process	biological process	1.83E-24	27
GO:0008061	chitin binding	molecular function	1.48E-23	26
GO:0006040	amino sugar metabolic process	biological process	2.81E-22	27
GO:0097367	carbohydrate derivative binding	molecular function	2.97E-21	26
GO:0006022	aminoglycan metabolic process	biological process	1.79E-20	28
GO:0004553	hydrolase activity, hydrolyzing O-glycosyl compounds	molecular function	1.47E-13	28
GO:0016798	hydrolase activity, acting on glycosyl bonds	molecular function	2.07E-12	28
GO:0005975	carbohydrate metabolic process	biological process	8.97E-08	39
GO:0005576	extracellular region	cellular	1.16E-05	42

		component		
GO:0004568	chitinase activity	molecular function	0.000805	5
GO:0006032	chitin catabolic process	biological process	0.000805	5
GO:0046348	amino sugar catabolic process	biological process	0.000805	5
GO:1901072	glucosamine-containing compound catabolic process	biological process	0.000805	5
GO:0016787	hydrolase activity	molecular function	0.000981	74
GO:1901135	carbohydrate derivative metabolic process	biological process	0.002228	33
GO:0016998	cell wall macromolecule catabolic process	biological process	0.007146	5
Af vs Cs				
GO:0006030	chitin metabolic process	biological process	4.45E-16	23
GO:1901071	glucosamine-containing compound metabolic process	biological process	4.45E-16	23
GO:0008061	chitin binding	molecular function	2.93E-15	22
GO:0006040	amino sugar metabolic process	biological process	2.33E-14	23
GO:0097367	carbohydrate derivative binding	molecular function	1.9E-13	22
GO:0006022	aminoglycan metabolic process	biological process	6.4E-13	24
GO:0008236	serine-type peptidase activity	molecular function	3.36E-10	29
GO:0017171	serine hydrolase activity	molecular function	3.36E-10	29
GO:0004553	hydrolase activity, hydrolyzing O-glycosyl compounds	molecular function	1.55E-07	24
GO:0070011	peptidase activity, acting on L-amino acid peptides	molecular function	3.2E-07	43
GO:0008233	peptidase activity	molecular function	1.01E-06	44
GO:0005576	extracellular region	cellular component	1.15E-06	53
GO:0016798	hydrolase activity, acting on glycosyl bonds	molecular function	1.15E-06	24
GO:0006508	proteolysis	biological process	1.29E-06	42
GO:0004252	serine-type endopeptidase activity	molecular function	2.91E-06	19
GO:0016787	hydrolase activity	molecular	5.93E-05	96

		function		
GO:0005975	carbohydrate metabolic process	biological process	0.001148	37
GO:0030880	RNA polymerase complex	cellular component	0.001927	13
GO:0004175	endopeptidase activity	molecular function	0.003418	28
GO:0005212	structural constituent of eye lens	molecular function	0.004515	3
GO:0016591	DNA-directed RNA polymerase II, holoenzyme	cellular component	0.012152	7
GO:0000428	DNA-directed RNA polymerase complex	cellular component	0.015093	7
GO:0055029	nuclear DNA-directed RNA polymerase complex	cellular component	0.015093	7
GO:0044767	single-organism developmental process	biological process	0.015093	11
GO:0030570	pectate lyase activity	molecular function	0.049087	2
Cs vs Lc				
GO:0006030	chitin metabolic process	biological process	1.9E-32	37
GO:1901071	glucosamine-containing compound metabolic process	biological process	1.9E-32	37
GO:0008061	chitin binding	molecular function	1.12E-31	36
GO:0006040	amino sugar metabolic process	biological process	2.96E-29	37
GO:0097367	carbohydrate derivative binding	molecular function	2.79E-28	36
GO:0006022	aminoglycan metabolic process	biological process	2.96E-27	39
GO:0042302	structural constituent of cuticle	molecular function	2.72E-17	22
GO:0008236	serine-type peptidase activity	molecular function	9.52E-10	31
GO:0017171	serine hydrolase activity	molecular function	9.52E-10	31
GO:0005576	extracellular region	cellular component	3.29E-09	66
GO:0070011	peptidase activity, acting on L-amino acid peptides	molecular function	3.52E-09	52
GO:0008233	peptidase activity	molecular function	6.04E-09	54
GO:0016798	hydrolase activity, acting on glycosyl bonds	molecular function	1.75E-08	29
GO:0004553	hydrolase activity, hydrolyzing O-	molecular	3.19E-08	27

	glycosyl compounds	function		
GO:0004252	serine-type endopeptidase activity	molecular function	3.01E-07	22
GO:0006508	proteolysis	biological process	4.31E-07	48
GO:1901135	carbohydrate derivative metabolic process	biological process	1.3E-05	51
GO:0016787	hydrolase activity	molecular function	1.42E-05	113
GO:0005975	carbohydrate metabolic process	biological process	1.53E-05	46
GO:0010466	negative regulation of peptidase activity	biological process	6.27E-05	5
GO:0051346	negative regulation of hydrolase activity	biological process	6.27E-05	5
GO:0004175	endopeptidase activity	molecular function	9.17E-05	35
GO:0043086	negative regulation of catalytic activity	biological process	0.000849	5
GO:0044092	negative regulation of molecular function	biological process	0.00093	5
GO:0030414	peptidase inhibitor activity	molecular function	0.000994	11
GO:0061134	peptidase regulator activity	molecular function	0.000994	11
GO:0052547	regulation of peptidase activity	biological process	0.002095	5
Cs vs Sk				
GO:0006030	chitin metabolic process	biological process	3.6E-28	36
GO:1901071	glucosamine-containing compound metabolic process	biological process	3.6E-28	36
GO:0008061	chitin binding	molecular function	1.65E-27	35
GO:0006040	amino sugar metabolic process	biological process	3.7E-25	36
GO:0006022	aminoglycan metabolic process	biological process	3.7E-25	40
GO:0097367	carbohydrate derivative binding	molecular function	2.64E-24	35
GO:0008236	serine-type peptidase activity	molecular function	5.36E-17	44
GO:0017171	serine hydrolase activity	molecular function	5.36E-17	44
GO:0004553	hydrolase activity, hydrolyzing O-glycosyl compounds	molecular function	2.17E-16	41
GO:0016798	hydrolase activity, acting on glycosyl	molecular	1.02E-14	41

	bonds	function		
GO:0016787	hydrolase activity	molecular function	1.96E-13	158
GO:0070011	peptidase activity, acting on L-amino acid peptides	molecular function	6.94E-12	64
GO:0008233	peptidase activity	molecular function	7.51E-12	67
GO:0004252	serine-type endopeptidase activity	molecular function	6.66E-11	29
GO:0005576	extracellular region	cellular component	7.29E-10	76
GO:0006508	proteolysis	biological process	1.09E-08	58
GO:0005975	carbohydrate metabolic process	biological process	2.53E-07	57
GO:0004175	endopeptidase activity	molecular function	6.7E-06	42
GO:0071554	cell wall organization or biogenesis	biological process	0.000183	12
GO:1901135	carbohydrate derivative metabolic process	biological process	0.000285	55
GO:0006026	aminoglycan catabolic process	biological process	0.000398	9
GO:0004568	chitinase activity	molecular function	0.000538	6
GO:0006032	chitin catabolic process	biological process	0.000538	6
GO:0046348	amino sugar catabolic process	biological process	0.000538	6
GO:1901072	glucosamine-containing compound catabolic process	biological process	0.000538	6
GO:0044036	cell wall macromolecule metabolic process	biological process	0.000614	10
GO:0016998	cell wall macromolecule catabolic process	biological process	0.000679	7
GO:0003824	catalytic activity	molecular function	0.02323	247
Lc vs Sk				
GO:0006030	chitin metabolic process	biological process	1.28E-36	36
GO:1901071	glucosamine-containing compound metabolic process	biological process	1.28E-36	36
GO:0008061	chitin binding	molecular function	1.22E-35	35
GO:0006040	amino sugar metabolic process	biological process	1.73E-33	36
GO:0097367	carbohydrate derivative binding	molecular	2.6E-32	35

		function		
GO:0006022	aminoglycan metabolic process	biological process	3.63E-29	36
GO:0042302	structural constituent of cuticle	molecular function	2.73E-16	19
GO:0005576	extracellular region	cellular component	9.24E-11	56
GO:0004553	hydrolase activity, hydrolyzing O-glycosyl compounds	molecular function	3.09E-08	23
GO:0016798	hydrolase activity, acting on glycosyl bonds	molecular function	4.29E-08	24
GO:1901135	carbohydrate derivative metabolic process	biological process	4.2E-06	42
GO:0005975	carbohydrate metabolic process	biological process	0.000629	34
GO:0004568	chitinase activity	molecular function	0.001156	5
GO:0006032	chitin catabolic process	biological process	0.001156	5
GO:0046348	amino sugar catabolic process	biological process	0.001156	5
GO:1901072	glucosamine-containing compound catabolic process	biological process	0.001156	5
GO:0008236	serine-type peptidase activity	molecular function	0.007537	16
GO:0017171	serine hydrolase activity	molecular function	0.007537	16
GO:0016998	cell wall macromolecule catabolic process	biological process	0.00995	5
GO:1901564	organonitrogen compound metabolic process	biological process	0.00995	55
GO:0070011	peptidase activity, acting on L-amino acid peptides	molecular function	0.026589	28
GO:0008233	peptidase activity	molecular function	0.041146	29

All genes were at corrected P-Value < 0.05, |log2.Fold_change| >1.

Table 7. The software and parameters for Non-reference transcriptome assembly and analysis

Analysis	Software	versions	Parameters	Remarks
Assembly	Trinity	r20140413p1	min_kmer_cov:2, other parameters are default)	-
Gene function annotation	NCBI blast 2.2.28+	v2.2.28+	NR, NT, Swiss-Prot: e-value = 1e-5;KOG/COG: e-value = 1e-3	NR, NT, KOG/COG, Swiss-Prot
	KAAS	r140224	e-value = 1e-10	KEGG annotation
	hmmscan	HMMER 3	e-value = 0.01	Pfam annotation
	blast2go	b2g4pipe_v2.5	e-value = 1.0E-6	GO annotation
Comparison of quantitative	RSEM	v1.2.15	bowtie2 parameters mismatch 0	Comparison of quantitative with transcripts assembled by trinity
Differential expression analysis	DEGSeq	1.12.0	qvalue< 0.005 & |log2(foldchange)| > 1	DESeq was used for biological replicate samples, samples without biological replicates using DEGSeq; edgeR was used in particular cases
	DESeq	1.10.1		
	edgeR	3.0.8		
GO enrichment	GOSeq,topGO	1.10.0,2.10.0	Corrected P-Value < 0.05	-
KEGG enrichment	KOBAS	v2.0.12	Corrected P-Value < 0.05	-
Protein interaction	NCBI blast 2.2.28+	v2.2.28+	e-value = 1e-10	Via blast
Canonical Correspondence analysis	Canoco	4.5		

Table 8. Designed sequences of real-time PCR primers for the candidate genes

Candidate genes	Sequence of primers (5' to 3')		Length (bp)
Insect cuticle protein	Forward	GCAATGAGCACGTCCAACTTC	184
	Reverse	TAGGTGGCAGTGGTCCTGTGA	
Peritrophin-1	Forward	GCAGGTTCCTCAGCATTGTCT	131
	Reverse	GTCTGCGACTACGTGTGGAATG	
Lactase-phlorizin hydrolase	Forward	GGCTCGCAAGATAGCTGACAC	138
	Reverse	CTCAACTGGTTGCACGACTCC	
Negative elongation factor A	Forward	ATTCTGCGGTGCATAATTTGAG	99
	Reverse	TGCCTACCACGGCTGATACC	
β-ACTIN	Forward	CCCATCTATGAAGGTTACGC	150
	Reverse	CTTGATGTCACGGACGATTT	
